# The efficacy and safety of annulus fibrosus suture as adjuvant therapy for lumbar disc herniation: a systematic review and meta-analysis

**DOI:** 10.3389/fbioe.2025.1741738

**Published:** 2026-01-27

**Authors:** Wensi Ouyang, Guimei Guo, Yu Sun, Haobo Jiang, Long Chen, Shaofeng Yang

**Affiliations:** 1 Graduate School of Hunan University of Chinese Medicine, Changsha, China; 2 Changchun University of Chinese Medicine, Changchun, China; 3 Department of Orthopedics, The First Affiliated Hospital of Hunan University of Chinese Medicine, Changsha, China

**Keywords:** annulus fibrosus suture, lumbar disc herniation, meta-analysis, surger, systematic review

## Abstract

**Objective:**

Lumbar disc herniation (LDH) has demonstrated a rising prevalence in contemporary clinical practice, significantly compromising patients’ daily lives and potentially necessitating surgical intervention. While annulus fibrosus suture (AFS) techniques are increasingly incorporated into surgical protocols, current evidence remains inconclusive regarding their definitive clinical advantages.

**Methods:**

We performed a comprehensive search of eight databases from inception to September 2025 to identify published articles on AFS for LDH. Outcome measures encompassed operative time, incision length, blood loss, length of stay (LOS), visual analog scale (VAS) score, Japanese Orthopedic Association (JOA) score, Oswestry disability index (ODI) score, disc height, recurrence, and complication. The quality of the studies was analyzed using the RoB-2 and ROBINS-I tools. Statistical analyses were performed using RevMan 5.4 software and Stata 17 software. The GRADE approach was used to evaluate the certainty of evidence for outcomes.

**Results:**

A total of 58 studies encompassing 5,765 patients diagnosed with LDH. The control group had shorter operative time (MD = 4.85, 95% CI: 2.79 to 6.92, *P* < 0.00001) compared to the AFS group. There were no differences in terms of incision length, blood loss, LOS, JOA score, ODI score, and complication between the techniques. AFS group demonstrated a significant benefit over control group in terms of VAS score (MD = −0.24, 95% CI: −0.33 to −0.14, *P* < 0.00001), disc height (SMD = 1.36, 95% CI: 0.73 to 2.00, *P* < 0.0001), and recurrence (RR = 0.34, 95% CI: 0.27 to 0.42, *P* < 0.00001). The results of subgroup analysis showed that different study types and different follow-up times were a source of heterogeneity. The quality of evidence for outcome measures ranges from very low to moderate.

**Conclusion:**

Current evidence suggests that AFS therapy may be advantageous in improving clinical symptoms and may reduce postoperative recurrence. Due to limited data and low quality of evidence, additional large-scale, multicenter trials are needed to verify and strengthen these findings.

## Introduction

Lumbar disc herniation (LDH) is a prevalent degenerative spinal disorder. LDH is caused by intervertebral disc degeneration, annulus fibrosus rupture, and nucleus pulposus tissue protrusion, which irritates the nerve root to cause clinical symptoms like lumbar back pain, motor, and sensory deficits (Mao et al., 2025; Takahashi et al., 2025). LDH, which seriously affects patients’ daily lives and work, has a rising incidence due to socioeconomic development and lifestyle changes (Du et al., 2025; Huang et al., 2025). Moreover, the patient population is becoming younger, posing a heavy social burden (Jung et al., 2020; Zhang A. S. et al., 2023; Jin et al., 2025a; Lee J. S. et al., 2025).

Currently, LDH treatment includes conservative and surgical approaches. Compared to conservative treatment, surgery more rapidly reduces pain, improves quality of life, and yields better patient satisfaction (Hwang et al., 2025; Lee C. C. et al., 2025; Park et al., 2025). The key to surgical treatment is to accurately remove the herniated nucleus pulposus tissue to maximize the preservation of disc function and minimize annulus fibrosus damage. However, the annulus fibrosus, mainly composed of collagen fibers with limited self-repair ability, is often structurally compromised during surgery (Feng et al., 2025; Guo et al., 2025). Its repair mainly relies on scar formation, resulting in low post-healing strength. The annulus incision or rupture site becomes a stress weak point. Residual nucleus pulposus in the intervertebral disc can easily re-herniate from here, causing LDH recurrence (Li and Wang, 2025; Li et al., 2023). To lower this risk, surgeons often remove more of the surrounding nucleus pulposus. However, this accelerates intervertebral height loss and disc degeneration, which may destabilize the spinal structure. Studies identify the extent of annular injury as a key predictor of postoperative recurrence in lumbar disc herniation. Some studies also suggest that the degree of annular fibrosus injury is one of the key factors influencing postoperative recurrence (Li X. et al., 2016; Zhao R. et al., 2024; Kong et al., 2025; Samir et al., 2025). Hence, it is crucial to preserve normal disc tissue and repair the damaged annulus fibrosus during surgery.

Current annulus fibrosus repair research focuses on two aspects, which are biological repair and physical repair (Wang Z. et al., 2025; Xin et al., 2025; Zhang A. et al., 2023; Zhou D. et al., 2023). Biological repair, using cell therapy, gene therapy, and biological materials, aims to maximally restore its physiological function (Ongini et al., 2025; Tang et al., 2025). Nevertheless, clinical application is limited due to inadequate mechanical strength, complex manufacturing processes, and poor biocompatibility (Desai et al., 2024; Ying et al., 2023). Physical repair uses various methods to close the annulus fibrosus rupture to restore its structural integrity. Annular closure devices, now employed as implants, have demonstrated efficacy in reducing recurrence and reoperation rates (Bouma et al., 2019; Miller et al., 2020; Thomé et al., 2021; Dalal et al., 2025). However, there is still a need to consider the impact of associated financial costs, postoperative complications, and potential device loosening on patients (Wang X. et al., 2022; Wang et al., 2024; Zhu Y. et al., 2025). Studies indicate that annulus fibrosus suture (AFS) can close ruptures directly, promote intervertebral disc structure restoration, maintain disc mechanical properties, and reduce scar formation (Wang F. et al., 2025; Bateman et al., 2016). However, some scholars argue that the AFS technique cannot provide sufficient mechanical strength to resist intradiscal pressure and annular fiber tension stress (Ning et al., 2023; Tavakoli et al., 2020). Therefore, this study aims to summarize the existing evidence and evaluate the efficacy and safety of AFS as an adjuvant treatment for LDH, offering more evidence-based medicine for clinical surgery.

## Methods and materials

### Protocol resister

This systematic review and meta-analysis was conducted according to the Preferred Reporting Items for Systematic Reviews and Meta-Analyses guidelines (Page et al., 2021; Page et al., 2018). Meanwhile, the protocol for this systematic review and meta-analysis was registered and published in the PROSPERO database on 2 May 2025 (registration number CRD420251078737).

### Search strategy

Two reviewers conducted comprehensive searches on a total of eight electronic databases, including PubMed, Web of Science, EMBASE, Cochrane Library, Chinese National Knowledge Infrastructure, China Science and Technology Journal Database, Chinese WanFang Database, and Chinese Biomedical Literature Database. The searches cover the period from the inception date of the databases until 20 September 2025. No language or geographic restrictions were applied. In this study, a combination of Medical Subject Headings and free terms was searched, and the search terms were modified appropriately for different databases. Search strategies incorporated keywords such as “annulus fibrosus repair”, “annulus fibrosus”, “annulus fibrosus suture”, “lumbar disc herniation”, “lumbar disc protrusion”, “intervertebral disc displacement”, and “LDH”. Additionally, reference lists of the included articles, relevant reviews, and gray literature were examined to maximize research potential. Two reviewers assessed the eligibility of the articles based on their titles, abstracts, and keywords. The full search methods for each database are depicted in the Supplementary Material.

### Eligibility criteria

- Types of research: This study included all published randomized controlled trials (RCTs), cohort studies, and case-control studies.
- Types of participant: The study included participants who were clearly diagnosed with LDH, without restrictions on age, gender, ethnicity, nationality, geographical location, or duration of disease.
- Types of intervention: The treatment group received surgery combined with any type of AFS technique.
- Types of comparison: The control group received surgery alone.
- Outcome measures: The outcomes assessed were operative time, incision length, blood loss, length of stay (LOS), visual analog scale (VAS) score, Japanese Orthopedic Association (JOA) score, Oswestry disability index (ODI) score, disc height, recurrence, and complication.

### Exclusion criteria

- Literature contains overlapping data or multiple publications (defined as multiple publications reporting on the same patient from the same institution and time period; only the most comprehensive report was retained).
- Reviews, conference papers, case reports, editor responses, animal experiments, basic experimental studies, technical notes, and review articles.
- The data recorded in the literature are unknown (defined as essential numerical data for meta-analysis not provided, only presented in non-extractable graphical form, or described only qualitatively).

### Literature screening and data extraction

Two reviewers assessed the eligibility of the articles based on their titles, abstracts, and keywords. The reviewer conducted an in-depth evaluation of the entire manuscript to make a final determination. Any disagreements were resolved by discussion and consensus. When necessary, the third reviewer made the final decision. Two independent reviewer authors used a systematic data extraction template to identify key study characteristics. Key study characteristics were identified using a systematic data extraction template, including details such as author details, year of publication, study design, sample size, sex ratio, patient mean age, body mass index, intervention, mean follow-up time, outcome indicators, and technical description of each treatment. Key outcomes were extracted by two other reviewers for data synthesis.

### Risk of bias of individual studies

Two reviewers independently evaluated the methodological quality of the included literature. For RCTs, quality assessment was performed using the Cochrane Risk of Bias assessment tool, version 2 (RoB-2) (Sterne et al., 2019). Non-RCTs were evaluated using the Cochrane’s Risk of Bias In Non-Randomized Studies of Interventions (ROBINS-I) (Sterne et al., 2016). Disagreements between the authors were resolved in discussions. If no consensus could be reached, a third reviewer made the final decision.

### Quality of evidence assessment

We utilized the principles of the Grades of Recommendation Assessment, Development, and Evaluation (GRADE) system to evaluate the quality of evidence related to outcomes (Atkins et al., 2004; Guyatt et al., 2011). The system includes research design, heterogeneity, risk of bias, indirectness, imprecision, and publication bias. The rating of the study design was downgraded by one level if data from a mixed study design were used; the rating was not downgraded if data from RCTs were used. Two reviewers independently evaluated each domain for every outcome selected. In cases of disagreement, a third reviewer was consulted for resolution. All decisions regarding upgrading or downgrading the certainty of evidence were carefully documented to maintain transparency.

### Statistical analysis

Statistical analysis was conducted using RevMan 5.4 software (Cochrane Collaboration, Oxford, UK) and Stata 17 software (StataCorp, College Station, USA). The risk ratio (RR) was utilized for comparing binary data, while the mean difference (MD) or standardized mean difference (SMD) was used for continuous data comparisons. The heterogeneity of articles was assessed using the chi-square test and *I* ^*2*^ statistics. The *I* ^*2*^ value of less than 50% indicated that there was no significant heterogeneity, and therefore, meta-analyses were conducted using a fixed-effects model. Conversely, a random effects model was used for the meta-analysis. If *I* ^*2*^ exceeded 50%, a subgroup analysis was conducted based on the study design and length of follow-up to identify the source of heterogeneity. A sensitivity analysis was conducted by systematically excluding one study at a time to evaluate the robustness of the results. In addition, we conducted sensitivity analyses for adolescent populations and high-risk studies. Publication bias was evaluated through the funnel plot test and Egger’s test.

## Results

### Search selection

**FIGURE 1:**
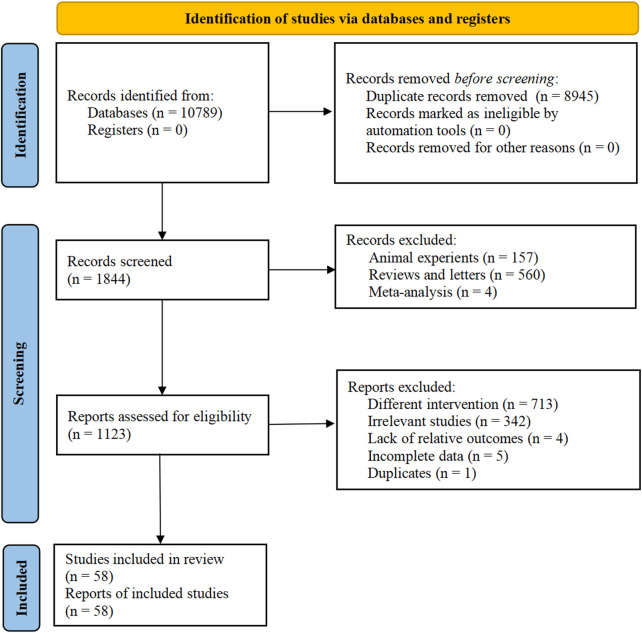
Literature screening process of the meta-.analysis.

A preliminary search of eight databases yielded an initial collection of 10,789 articles on AFS as an adjuvant therapy for LDH. After removing 8,945 duplicate records, 721 articles were excluded after skimming through the title and abstract. Furthermore, 1,065 articles were eliminated after a thorough review of the full texts against the inclusion and exclusion criteria. Consequently, a total of 58 published articles (Gao and Liang, 2025; He et al., 2025; Zhang Q. et al., 2025; Zhou et al., 2025; Chen et al., 2024; Fu et al., 2024; He et al., 2024; Hu and Chen, 2024; Li and Du, 2024; Liu et al., 2024; Peng et al., 2024; Shi et al., 2024; Sun et al., 2024; Xi et al., 2024; Yu et al., 2024; Zhao Y. F. et al., 2024; Liang et al., 2023; Liu et al., 2023; Song et al., 2023; Tian et al., 2023; Wu et al., 2023; Ding et al., 2022; Tang and Xu, 2022; Wang Z. et al., 2022; Xu et al., 2022; Yao et al., 2022; Jiao et al., 2021; Li J. et al., 2021; Xiao, 2021; Zhang L. et al., 2021; Chen, 2020; Fu et al., 2020; Ren et al., 2020; Song et al., 2020; Chen et al., 2019; Li, 2019; Yi et al., 2019; Zhang, 2019; He et al., 2018; Hu, 2018; Liu, 2018; Song et al., 2018; Xu et al., 2018) were included in this meta-analysis (Figure 1).

### Baseline characteristics

A total of 5,765 participants with LDH were included in the 58 studies (Gao and Liang, 2025; He et al., 2025; Zhang Q. et al., 2025; Zhou et al., 2025; Chen et al., 2024; Fu et al., 2024; He et al., 2024; Hu and Chen, 2024; Li and Du, 2024; Liu et al., 2024; Peng et al., 2024; Shi et al., 2024; Sun et al., 2024; Xi et al., 2024; Yu et al., 2024; Zhao Y. F. et al., 2024; Liang et al., 2023; Liu et al., 2023; Song et al., 2023; Tian et al., 2023; Wu et al., 2023; Ding et al., 2022; Tang and Xu, 2022; Wang Z. et al., 2022; Xu et al., 2022; Yao et al., 2022; Jiao et al., 2021; Li J. et al., 2021; Xiao, 2021; Zhang L. et al., 2021; Chen, 2020; Fu et al., 2020; Ren et al., 2020; Song et al., 2020; Chen et al., 2019; Li, 2019; Yi et al., 2019; Zhang, 2019; He et al., 2018; Hu, 2018; Liu, 2018; Song et al., 2018; Xu et al., 2018). There were 2,990 participants in the control group and 2,775 participants in the treatment group. All clinical trials were conducted from 2013 to 2025. The patients’ average age in the control group ranged from 14.63 to 69.81, while the average age of the treatment group ranged from 14.41 to 69.86. The follow-up period for the patients enrolled in these studies ranged from 6 months to 4.21 years. All studies had clear inclusion and exclusion criteria, with no significant baseline differences between the control and treatment groups. Among the 58 studies, forty-six investigated the effects of operative time (Gao and Liang, 2025; He et al., 2025; Zhang Q. et al., 2025; Zhou et al., 2025; Chen et al., 2024; Fu et al., 2024; He et al., 2024; Hu and Chen, 2024; Peng et al., 2024; Shi et al., 2024; Sun et al., 2024; Xi et al., 2024; Yu et al., 2024; Zhao Y. F. et al., 2024; Liu et al., 2023; Song et al., 2023; Tian et al., 2023; Ding et al., 2022; Tang and Xu, 2022; Wang Z. et al., 2022; Xu et al., 2022; Yao et al., 2022; Jiao et al., 2021; Xiao, 2021; Zhang L. et al., 2021; Chen, 2020; Ren et al., 2020; Song et al., 2020; Li, 2019; Zhang, 2019; He et al., 2018; Hu, 2018; Liu, 2018; Song et al., 2018; Guo et al., 2017; Jiang et al., 2017; Li et al., 2017; Lv, 2017; Yu et al., 2017; Zhang L. et al., 2017; Zhang G. G. et al., 2017; Zhang, 2017; Zhu G. H. et al., 2017), thirteen examined the effects of incision length (He et al., 2025; Zhang Q. et al., 2025; Hu and Chen, 2024; Shi et al., 2024; Liu et al., 2023; Jiao et al., 2021; Zhang L. et al., 2021; Song et al., 2018; Jiang et al., 2017; Li et al., 2017; Zhang L. et al., 2017; Zhang G. G. et al., 2017; Li J. et al., 2016), forty-one specifically explored the effects of blood loss (Gao and Liang, 2025; He et al., 2025; Zhang Q. et al., 2025; Zhou et al., 2025; Chen et al., 2024; He et al., 2024; Hu and Chen, 2024; Peng et al., 2024; Shi et al., 2024; Yu et al., 2024; Zhao Y. F. et al., 2024; Liu et al., 2023; Tian et al., 2023; Ding et al., 2022; Tang and Xu, 2022; Xu et al., 2022; Yao et al., 2022; Jiao et al., 2021; Xiao, 2021; Zhang L. et al., 2021; Chen, 2020; Song et al., 2020; Li, 2019; Zhang, 2019; He et al., 2018; Hu, 2018; Liu, 2018; Song et al., 2018; Xu et al., 2018; Guo et al., 2017; Jiang et al., 2017; Li et al., 2017; Lv, 2017; Yu et al., 2017; Zhang L. et al., 2017; Zhang G. G. et al., 2017; Zhang, 2017; Zhu G. H. et al., 2017; Zhu Z. Y. et al., 2017; Li J. et al., 2016; Li et al., 2014), twenty-three reported LOS (Zhang Q. et al., 2025; Zhou et al., 2025; Fu et al., 2024; Hu and Chen, 2024; Li and Du, 2024; Peng et al., 2024; Shi et al., 2024; Yu et al., 2024; Liu et al., 2023; Tang and Xu, 2022; Wang Z. et al., 2022; Xu et al., 2022; Jiao et al., 2021; Xiao, 2021; Zhang L. et al., 2021; Ren et al., 2020; Song et al., 2018; Guo et al., 2017; Li et al., 2017; Lv, 2017; Zhang L. et al., 2017; Zhang G. G. et al., 2017; Li J. et al., 2016), forty-seven trials focused on the impact of VAS score (Gao and Liang, 2025; Zhang Q. et al., 2025; Zhou et al., 2025; Fu et al., 2024; He et al., 2024; Hu and Chen, 2024; Li and Du, 2024; Liu et al., 2024; Peng et al., 2024; Shi et al., 2024; Sun et al., 2024; Xi et al., 2024; Yu et al., 2024; Zhao Y. F. et al., 2024; Liang et al., 2023; Liu et al., 2023; Song et al., 2023; Wu et al., 2023; Ding et al., 2022; Tang and Xu, 2022; Wang Z. et al., 2022; Yao et al., 2022; Li J. et al., 2021; Xiao, 2021; Zhang L. et al., 2021; Chen, 2020; Fu et al., 2020; Ren et al., 2020; Song et al., 2020; Chen et al., 2019; Li, 2019; Yi et al., 2019; Zhang, 2019; He et al., 2018; Hu, 2018; Song et al., 2018; Xu et al., 2018; Jiang et al., 2017; Li et al., 2017; Lv, 2017; Yu et al., 2017; Zhang L. et al., 2017; Zhang, 2017), twelve investigated the effects of JOA score (Zhou et al., 2025; He et al., 2024; Hu and Chen, 2024; Shi et al., 2024; Tang and Xu, 2022; Jiao et al., 2021; Fu et al., 2020; Song et al., 2018; Yu et al., 2017; Zhu G. H. et al., 2017; Zhang et al., 2015; Cai et al., 2013), forty-four examined the effects of ODI score (Gao and Liang, 2025; Zhang Q. et al., 2025; Chen et al., 2024; Fu et al., 2024; He et al., 2024; Hu and Chen, 2024; Li and Du, 2024; Liu et al., 2024; Peng et al., 2024; Shi et al., 2024; Sun et al., 2024; Xi et al., 2024; Yu et al., 2024; Liang et al., 2023; Liu et al., 2023; Song et al., 2023; Wu et al., 2023; Ding et al., 2022; Tang and Xu, 2022; Wang Z. et al., 2022; Yao et al., 2022; Li J. et al., 2021; Xiao, 2021; Zhang L. et al., 2021; Chen, 2020; Fu et al., 2020; Ren et al., 2020; Song et al., 2020; Chen et al., 2019; Li, 2019; Zhang, 2019; He et al., 2018; Hu, 2018; Song et al., 2018; Xu et al., 2018; Jiang et al., 2017; Li et al., 2017; Lv, 2017; Yu et al., 2017; Zhang L. et al., 2017; Zhang, 2017; Zhu Z. Y. et al., 2017; Li J. et al., 2016), eleven explored the effects of disc height (Shi et al., 2024; Xi et al., 2024; Liu et al., 2023; Yao et al., 2022; Chen, 2020; Fu et al., 2020; Ren et al., 2020; Li, 2019; Zhang, 2019; Zhu Z. Y. et al., 2017; Zhang et al., 2015), forty-eight specifically examined the effects of recurrence (Gao and Liang, 2025; He et al., 2025; Zhang Q. et al., 2025; Zhou et al., 2025; Chen et al., 2024; Fu et al., 2024; He et al., 2024; Hu and Chen, 2024; Li and Du, 2024; Liu et al., 2024; Peng et al., 2024; Shi et al., 2024; Xi et al., 2024; Yu et al., 2024; Zhao Y. F. et al., 2024; Liang et al., 2023; Liu et al., 2023; Song et al., 2023; Tian et al., 2023; Wu et al., 2023; Ding et al., 2022; Tang and Xu, 2022; Wang Z. et al., 2022; Xu et al., 2022; Yao et al., 2022; Li J. et al., 2021; Xiao, 2021; Zhang L. et al., 2021; Chen, 2020; Ren et al., 2020; Song et al., 2020; Chen et al., 2019; Li, 2019; Zhang, 2019; He et al., 2018; Hu, 2018; Liu, 2018; Xu et al., 2018; Jiang et al., 2017; Zhang L. et al., 2017; Zhang G. G. et al., 2017; Zhang, 2017; Zhu G. H. et al., 2017), and nine focused on the impact of complication (Zhou et al., 2025; Li and Du, 2024; Shi et al., 2024; Ren et al., 2020; Jiang et al., 2017; Li et al., 2017; Zhang G. G. et al., 2017; Zhu G. H. et al., 2017; Bailey et al., 2013). Tables 1–3 summarize the characteristics of the included studies.

**TABLE 1:** Basic characteristics of the fifty-eight studies included in the meta-analysis.

Inclusion studies	Study design	Sample size (Male/Female)	Patient age (years)	BMI (kg/m2)	Disease duration (months)
Gao and Liang (2025)	Cohort study	T: 26 (18/8) C: 32 (22/10)	T: 45 ± 12 C: 46 ± 10	T: NA C: NA	T: NA C: NA
He et al. (2025)	RCT	T: 37 (22/15) C: 37 (21/16)	T: 15.51 ± 3.02 C: 15.26 ± 3.08	T: NA C: NA	T: 11.36 ± 2.11 Month C: 11.26 ± 2.04 Month
Zhang et al. (2025a)	Cohort study	T: 22 (14/8) C: 34 (22/12)	T: 41.8 ± 10.6 C: 43.4 ± 9.8	T: 23.1 ± 2.8 C: 22.7 ± 2.6	T: 13.4 ± 2.4 Month C: 13.0 ± 2.6 Month
Zhou et al. (2025)	Cohort study	T: 39 (13/26) C: 138 (69/69)	T: 57.98 ± 13.65 C: 59.26 ± 12.16	T: 24.21 ± 3.26 C: 24.53 ± 3.20	T: NA C: NA
Chen et al. (2024)	Cohort study	T: 35 (21/14) C: 31 (20/11)	T: 51.48 ± 9.33 C: 47.94 ± 8.81	T: NA C: NA	T: 12.45 ± 2.13 Month C: 11.58 ± 2.67 Month
Fu et al. (2024)	Cohort study	T: 44 (26/18) C: 42 (25/17)	T: 38.8 ± 5.2 C: 37.9 ± 4.6	T: 26.38 ± 4.21 C: 26.18 ± 4.59	T: NA C: NA
He et al. (2024)	Cohort study	T: 212 (NA) C: 208 (NA)	39.9 ± 5.1	T: NA C: NA	T: NA C: NA
Hu and Chen (2024)	RCT	T: 40 (29/11) C: 40 (28/12)	T: 69.86 ± 3.38 C: 69.81 ± 3.57	T: 21.55 ± 3.06 C: 21.68 ± 3.12	T: NA C: NA
Li and Du (2024)	Cohort study	T: 25 (13/12) C: 25 (14/11)	T: 65.16 ± 2.32 C: 64.27 ± 2.41	T: NA C: NA	T: NA C: NA
Liu et al. (2024)	Cohort study	T: 35 (19/16) C: 47 (25/22)	T: 48.32 ± 6.92 C: 47.65 ± 6.00	T: NA C: NA	T: NA C: NA
Peng et al. (2024)	Cohort study	T: 31 (15/16) C: 41 (28/13)	T: 35.7 ± 7.2 C: 36.0 ± 12.2	T: 22.5 ± 1.9 C: 22.9 ± 1.8	T: 13.1 ± 5.5 Month C: 14.7 ± 5.6 Month
Shi et al. (2024)	Cohort study	T: 58 (29/29) C: 59 (33/26)	T: 49.31 ± 5.47 C: 48.25 ± 5.36	T: 22.42 ± 1.24 C: 22.74 ± 1.26	T: 16.85 ± 3.37 Month C: 17.56 ± 3.51 Month
Sun et al. (2024)	Cohort study	T: 60 (34/26) C: 67 (41/26)	T: 25.3 ± 2.9 C: 24.4 ± 3.5	T: 24.6 ± 3.3 C: 24.3 ± 3.1	T: NA C: NA
Xi et al. (2024)	Cohort study	T: 33 (15/18) C: 73 (31/42)	T: 47.81 ± 11.61 C: 52.81 ± 9.45	T: 22.91 ± 3.61 C: 23.66 ± 2.79	T: NA C: NA
Yu et al. (2024)	Cohort study	T: 42 (22/20) C: 41 (18/23)	T: 44.62 ± 7.02 C: 45.83 ± 7.31	T: NA C: NA	T: 10.21 ± 5.15 Month C: 10.31 ± 5.56 Month
Zhao et al. (2024b)	Cohort study	T: 46 (26/20) C: 50 (22/28)	T: 36.78 ± 7.01 C: 34.56 ± 7.04	T: NA C: NA	T: NA C: NA
Liang et al. (2023)	Cohort study	T: 27 (15/12) C: 32 (18/14)	T: NA C: NA	T: NA C: NA	T: NA C: NA
Liu et al. (2023)	Cohort study	T: 35 (19/16) C: 47 (25/22)	T: 48.3 ± 6.9 C: 47.7 ± 6.0	T: 22.5 ± 1.1 C: 22.6 ± 1.3	T: 8.1 ± 2.0 Month C: 7.7 ± 2.5 Month
Song et al. (2023)	Cohort study	T: 30 (23/7) C: 30 (22/8)	T: 46.2 ± 6.64 C: 45.83 ± 8.24	T: 22.64 ± 3.15 C: 23.17 ± 2.96	T: NA C: NA
Tian et al. (2023)	RCT	T: 40 (22/18) C: 40 (21/19)	T: 60.3 ± 15.2 C: 61.5 ± 15.5	T: NA C: NA	T: 22.2 ± 2.4 Month C: 22.4 ± 2.1 Month
Wu et al. (2023)	Cohort study	T: 37 (21/16) C: 30 (18/12)	T: 14.41 ± 1.89 C: 14.63 ± 1.95	T: NA C: NA	T: 10.05 ± 2.57 Month C: 10.36 ± 2.74 Month
Ding et al. (2022)	Cohort study	T: 38 (25/13) C: 45 (24/21)	T: 39.05 ± 14.11 C: 43.13 ± 16.62	T: NA C: NA	T: 13.76 ± 5.68 Month C: 14.93 ± 5.73 Month
Tang and Xu (2022)	Cohort study	T: 32 (20/12) C: 36 (23/13)	T: 45.87 ± 6.11 C: 44.98 ± 6.31	T: NA C: NA	T: NA C: NA
Wang et al. (2022b)	Cohort study	T: 40 (22/18) C: 42 (23/19)	T: NA C: NA	T: NA C: NA	T: NA C: NA
Xu et al. (2022)	Cohort study	T: 32 (18/14) C: 33 (18/15)	T: 43 ± 6.8 C: 46 ± 9.3	T: NA C: NA	T: NA C: NA
Yao et al. (2022)	Cohort study	T: 35 (19/16) C: 47 (25/22)	T: 36.2 ± 13.2 C: 38.2 ± 13.6	T: NA C: NA	T: NA C: NA
Jiao et al. (2021)	Cohort study	T: 39 (21/18) C: 47 (26/21)	T: 15.3 ± 1.9 C: 16.9 ± 0.8	T: NA C: NA	T: NA C: NA
Li et al. (2021a)	RCT	T: 25 (NA) C: 25 (NA)	T: NA C: NA	T: NA C: NA	T: NA C: NA
Xiao (2021)	RCT	T: 100 (54/46) C: 100 (56/44)	T: 46.46 ± 8.74 C: 46.23 ± 8.48	T: NA C: NA	T: 4.34 ± 0.98 Month C: 4.37 ± 0.92 Month
Zhang et al. (2021a)	Cohort study	T: 10 (7/3) C: 12 (8/4)	T: 19.3 ± 1.5 C: 19.2 ± 1.5	T: NA C: NA	T: NA C: NA
Chen (2020)	RCT	T: 60 (29/31) C: 60 (32/28)	T: 47.31 ± 10.52 C: 46.52 ± 10.38	T: NA C: NA	T: NA C: NA
Fu et al. (2020)	Cohort study	T: 31 (18/13) C: 25 (16/9)	T: 29.45 ± 7.14 C: 27.78 ± 5.47	T: 22.08 ± 1.69 C: 21.77 ± 1.54	T: 10.40 ± 2.49 Month C: 11.13 ± 2.36 Month
Ren et al. (2020)	Cohort study	T: 51 (NA) C: 54 (NA)	T: 42.0 ± 11.6 C: 45.6 ± 12.2	T: NA C: NA	T: 17.9 ± 4.1 Week C: 19.6 ± 4.7 Week
Song et al. (2020)	Cohort study	T: 35 (23/12) C: 41 (26/15)	T: 42.39 ± 10.66 C: 37.5 ± 12.7	T: NA C: NA	T: NA C: NA
Chen et al. (2019)	Cohort study	T: 20 (NA) C: 20 (NA)	T: NA C: NA	T: NA C: NA	T: NA C: NA
Li (2019)	Case-control study	T: 42 (25/17) C: 55 (31/24)	T: 42.3 ± 9.7 C: 41.8 ± 7.6	T: NA C: NA	T: 3.2 ± 0.5 Year C: 3.4 ± 0.6 Year
Yi et al. (2019)	Cohort study	T: 22 (18/4) C: 28 (22/6)	T: 38 ± 17 C: 42 ± 10	T: NA C: NA	T: NA C: NA
Zhang (2019)	Cohort study	T: 39 (22/17) C: 44 (26/18)	T: 42.6 ± 9.3 C: 44.1 ± 7.5	T: NA C: NA	T: 37.2 ± 6.5 Month C: 35.8 ± 4.6 Month
He et al. (2018)	RCT	T: 21 (12/9) C: 24 (13/11)	T: 35 ± 10 C: 34 ± 11	T: NA C: NA	T: NA C: NA
Hu (2018)	Cohort study	T: 39 (22/17) C: 37 (23/14)	T: 42.5 ± 8.6 C: 43.7 ± 9.1	T: NA C: NA	T: NA C: NA
Liu (2018)	RCT	T: 60 (35/25) C: 60 (31/29)	T: 50.9 ± 7.2 C: 51.3 ± 7.2	T: NA C: NA	T: 2.9 ± 0.4 Year C: 3.0 ± 0.4 Year
Song et al. (2018)	Cohort study	T: 20 (12/8) C: 16 (9/7)	T: 16.9 ± 1.2 C: 17.4 ± 1.5	T: NA C: NA	T: NA C: NA
Xu et al. (2018)	Cohort study	T: 33 (20/13) C: 33 (19/14)	T: 46.19 ± 6.35 C: 47.28 ± 7.41	T: NA C: NA	T: 3.83 ± 1.17 Year C: 3.67 ± 1.05 Year
Guo et al. (2017)	Cohort study	T: 28 (17/11) C: 31 (19/12)	T: 49.52 ± 4.38 C: 49.48 ± 4.35	T: NA C: NA	T: 3.2 ± 0.7 Month C: 3.2 ± 0.8 Month
Jiang et al. (2017)	RCT	T: 25 (12/13) C: 23 (11/12)	T: 48.68 ± 6.00 C: 49.91 ± 7.01	T: NA C: NA	T: NA C: NA
Li et al. (2017)	Cohort study	T: 32 (19/13) C: 32 (20/12)	T: 39.03 ± 11.36 C: 38.96 ± 11.03	T: NA C: NA	T: 5.71 ± 2.68 Year C: 5.59 ± 2.58 Year
Lv (2017)	RCT	T: 34 (23/11) C: 34 (21/13)	T: 45.28 ± 2.94 C: 46.25 ± 3.27	T: NA C: NA	T: 2.69 ± 0.82 Year C: 2.36 ± 0.67 Year
Yu et al. (2017)	Cohort study	T: 30 (13/17) C: 30 (14/16)	T: 39.8 ± 11.1 C: 40.3 ± 10.2	T: NA C: NA	T: NA C: NA
Zhang et al. (2017a)	RCT	T: 28 (17/11) C: 28 (19/9)	T: 46.1 ± 11.4 C: 44.9 ± 12.5	T: NA C: NA	T: 4.3 ± 0.9 Month C: 4.1 ± 1.2 Month
Zhang et al. (2017b)	Cohort study	T: 39 (21/18) C: 47 (24/23)	T: 14.3 ± 1.5 C: 15.1 ± 1.8	T: NA C: NA	T: NA C: NA
Zhang (2017)	RCT	T: 36 (19/17) C: 33 (19/14)	T: 45.2 ± 4.7 C: 43.8 ± 5.1	T: NA C: NA	T: NA C: NA
Zhu et al. (2017a)	Cohort study	T: 46 (NA) C: 46 (NA)	51.7 ± 8.2	T: NA C: NA	13.9 ± 1.8 Month
Zhu et al. (2017b)	Cohort study	T: 118 (63/55) C: 172 (97/75)	T: 35.9 ± 11.6 C: 39.1 ± 12.2	T: NA C: NA	T: NA C: NA
Li et al. (2016b)	Cohort study	T: 12 (7/5) C: 14 (9/5)	T: 17.9 ± 1.3 C: 18.2 ± 1.8	T: NA C: NA	T: NA C: NA
Zhang et al. (2015)	Cohort study	T: 18 (14/4) C: 20 (12/8)	T: 28.2 ± 6.4 C: 26.9 ± 6.1	T: NA C: NA	T: 9.9 ± 3.9 Month C: 10 ± 2.8 Month
Li et al. (2014)	RCT	T: 51 (30/21) C: 168 (92/76)	T: 38.8 ± 11.7 C: 37.7 ± 12.4	T: NA C: NA	T: NA C: NA
Bailey et al. (2013)	RCT	T: 478 (284/194) C: 249 (140/109)	T: 42.4 ± 11.3 C: 41.9 ± 11.6	T: 28.6 ± 5.3 C: 29.1 ± 6.2	T: 513 ± 1,101 Day C: 487 ± 1,119 Day
Cai et al. (2013)	Cohort study	T: 22 (17/5) C: 35 (23/12)	T: NA C: NA	T: NA C: NA	T: NA C: NA

C, control group; NA, not available; RCT, randomized controlled trial; T, treatment group.

**TABLE 2:** Intervention characteristics of included studies.

Inclusion studies	Treatment group	Control group	Suture equipment	Responsible levels	Outcomes	Follow-up
Gao and Liang (2025)	AFS + PTED	PTED	Disposable Suture Device	T: L4/L5 = 12, L5/S1 = 14 C: L3/L4 = 1 L4/L5 = 14, L5/S1 = 17	Operative time Blood loss VAS score ODI score Recurrence	15.7 Month
He et al. (2025)	AFS + PTED	PTED	Disposable Suture Device (EFIT-Ⅱ)	T: L3/L4 = 14, L4/L5 = 16, L5/S1 = 6 C: L3/L4 = 15, L4/L5 = 13, L5/S1 = 9	Operative time Incision length Blood loss Recurrence	12 Month
Zhang et al. (2025a)	AFS + PEID	TELD	Disposable Suture Device	T: L3/L4 = 5, L4/L5 = 9, L5/S1 = 8 C: L3/L4 = 8, L4/L5 = 14, L5/S1 = 12	Operative time Incision length Blood loss LOS VAS score ODI score Recurrence	14.3 ± 0.2 Month
Zhou et al. (2025)	AFS + MED	MED	Disposable Suture Device	NA	Operative time Blood loss LOS VAS score JOA score Recurrence Complication	T: 26.46 ± 2.01 Month C: 26.83 ± 2.68 Month
Chen et al. (2024)	AFS + PELD	PELD	Disposable Suture Device	T: L3/L4 = 9, L4/L5 = 20, L5/S1 = 6 C: L3/L4 = 8, L4/L5 = 17, L5/S1 = 6	Operative time Blood loss VAS score ODI score Recurrence	12–15 Month
Fu et al. (2024)	AFS + PELD	PELD	Disposable Suture Device (STAR-S)	T: L3/L4 = 3, L4/L5 = 22, L5/S1 = 19 C: L3/L4 = 2, L4/L5 = 21, L5/S1 = 19	Operative time LOS VAS score ODI score Recurrence	12 Month
He et al. (2024)	AFS + PELD	PELD	NA	NA	Operative time Blood loss VAS score JOA score ODI score Recurrence	15.2 ± 1.6 Month
Hu and Chen (2024)	AFS + PTED	PTED	Disposable Suture Device	T: L4/L5 = 21, L5/S1 = 10, other = 9 C: L4/L5 = 20, L5/S1 = 11, other = 9	Operative time Incision length Blood loss LOS VAS score JOA score ODI score Recurrence	12 Month
Li and Du (2024)	AFS + AUSS	AUSS	Disposable Suture Device	NA	LOS VAS score ODI score Recurrence Complication	NA
Liu et al. (2024)	AFS + PELD	PELD	Disposable Suture Device	T: L4/L5 = 20, L5/S1 = 15 C: L4/L5 = 27, L5/S1 = 20	VAS score ODI score Recurrence	18 Month
Peng et al. (2024)	AFS + TELD	TELD	Disposable Suture Device	T: L4/L5 = 11, L5/S1 = 20 C: L4/L5 = 16, L5/S1 = 25	Operative time Blood loss LOS VAS score ODI score Recurrence	12–24 Month
Shi et al. (2024)	AFS + PTED	PTED	Disposable Suture Device	T: L4/L5 = 34, L5/S1 = 24 C: L4/L5 = 30, L5/S1 = 29	Operative time Incision length Blood loss LOS VAS score JOA score ODI score Disc height Recurrence Complication	12 Month
Sun et al. (2024)	AFS + PEID	PEID	Disposable Suture Device	T: L4/L5 = 37, L5/S1 = 23 C: L4/L5 = 42, L5/S1 = 25	Operative time VAS score ODI score	12 Month
Xi et al. (2024)	AFS + PELD	PELD	Disposable Suture Device	T: L3/L4 = 10, L4/L5 = 13, L5/S1 = 10 C: L3/L4 = 17, L4/L5 = 35, L5/S1 = 21	Operative time VAS score ODI score Disc height Recurrence	12 Month
Yu et al. (2024)	AFS + MED	MED	Disposable Suture Device	T: L3/L4 = 6, L4/L5 = 21, L5/S1 = 15 C: L3/L4 = 4, L4/L5 = 20, L5/S1 = 17	Operative time Blood loss LOS VAS score ODI score Recurrence	T: 14.13 ± 0.92 Month C: 13.31 ± 1.31 Month
Zhao et al. (2024b)	AFS + PTED	PTED	Disposable Suture Device	T: L2/L3 = 1, L3/L4 = 11, L4/L5 = 16, L5/S1 = 18 C: L2/L3 = 2, L3/L4 = 14, L4/L5 = 17, L5/S1 = 17	Operative time Blood loss VAS score Recurrence	12 Month
Liang et al. (2023)	AFS + MED	MED	NA	NA	VAS score ODI score Recurrence	12 Month
Liu et al. (2023)	AFS + PELD	PELD	Disposable Suture Device	T: L4/L5 = 20, L5/S1 = 15 C: L4/L5 = 27, L5/S1 = 20	Operative time Incision length Blood loss LOS VAS score ODI score Disc height Recurrence	18 Month
Song et al. (2023)	AFS + TELD	TELD	Disposable Suture Device	T: L4/L5 = 12, L5/S1 = 18 C: L4/L5 = 10, L5/S1 = 20	Operative time VAS score ODI score Recurrence	15 ± 8 Month
Tian et al. (2023)	AFS + Discectomy	Discectomy	Disposable Suture Device	NA	Operative time Blood loss Recurrence	6 Month
Wu et al. (2023)	AFS + PTED	PTED	Disposable Suture Device (EFIT-Ⅱ)	T: L3/L4 = 6, L4/L5 = 20, L5/S1 = 11 C: L3/L4 = 5, L4/L5 = 17, L5/S1 = 8	VAS score ODI score Recurrence	14.2 ± 1.7 Month
Ding et al. (2022)	AFS + TELD	TELD	Disposable Suture Device	T: L4/L5 = 15, L5/S1 = 23 C: L4/L5 = 26, L5/S1 = 19	Operative time Blood loss VAS score ODI score Recurrence	16.82 ± 3.75 Month
Tang and Xu (2022)	AFS + MED	MED	Disposable Suture Device	T: L3/L4 = 5, L4/L5 = 14, L5/S1 = 13 C: L3/L4 = 4, L4/L5 = 18, L5/S1 = 14	Operative time Blood loss LOS VAS score JOA score ODI score Recurrence	4.21 ± 0.54 Year
Wang et al. (2022b)	AFS + TELD	TELD	Disposable Suture Device	T: L3/L4 = 1, L4/L5 = 20, L5/S1 = 19 C: L3/L4 = 3, L4/L5 = 22, L5/S1 = 17	Operative time LOS VAS score ODI score Recurrence	18 Month
Xu et al. (2022)	AFS + MED	MED	Disposable Suture Device	T: L3/L4 = 3 L4/L5 = 19 L5/S1 = 10 C: L3/L4 = 3 L4/L5 = 15 L5/S1 = 15	Operative time Blood loss LOS Recurrence	NA
Yao et al. (2022)	AFS + Discectomy	Discectomy	Disposable Suture Device (FAST-FIX)	T: L4/L5 = 20, L5/S1 = 15 C: L4/L5 = 27, L5/S1 = 20	Operative time Blood loss VAS score ODI score Disc height Recurrence	24 Month
Jiao et al. (2021)	AFS + OD	OD	Disposable Suture Device	T: L4/L5 = 23, L5/S1 = 16 C: L4/L5 = 33, L5/S1 = 14	Operative time Incision length Blood loss LOS JOA score	NA
Li et al. (2021a)	AFS + TELD	TELD	Disposable Suture Device	L3/L4 = 15 L4/L5 = 37, L5/S1 = 23	VAS score ODI score Recurrence	6 Month
Xiao (2021)	AFS + Discectomy	Discectomy	Disposable Suture Device (FAST-FIX)	NA	Operative time Blood loss LOS VAS score ODI score Recurrence	6 Month
Zhang et al. (2021a)	AFS + Discectomy	Discectomy	Disposable Suture Device	T: L4/L5 = 4, L5/S1 = 6 C: L4/L5 = 9, L5/S1 = 3	Operative time Incision length Blood loss LOS VAS score ODI score Recurrence	21.3 ± 7.6 Month
Chen (2020)	AFS + MED	MED	Disposable Suture Device	T: L3/L4 = 23, L4/L5 = 24, L5/S1 = 13 C: L3/L4 = 21, L4/L5 = 23, L5/S1 = 16	Operative time Blood loss VAS score ODI score Disc height Recurrence	6 Month
Fu et al. (2020)	AFS + OD	OD	Disposable Suture Device	NA	VAS score JOA score ODI score Disc height	T: 13.5 Month C: 15 Month
Ren et al. (2020)	AFS + MED	PTED	Disposable Suture Device	T: L3/L4 = 6, L4/L5 = 26, L5/S1 = 19 C: L3/L4 = 6, L4/L5 = 25, L5/S1 = 23	Operative time LOS VAS score ODI score Disc height Recurrence Complication	36 Month
Song et al. (2020)	AFS + PELD	PELD	NA	NA	Operative time Blood loss VAS score ODI score Recurrence	16.1 Month
Chen et al. (2019)	AFS + Discectomy	Discectomy	Disposable Suture Device	NA	VAS score ODI score Recurrence	12 Month
Li (2019)	AFS + MED	MED	Disposable Suture Device (FAST-FIX)	T: L3/L4 = 3, L4/L5 = 24, L5/S1 = 15 C: L3/L4 = 5, L4/L5 = 31, L5/S1 = 19	Operative time Blood loss VAS score ODI score Disc height Recurrence	27.1 Month
Yi et al. (2019)	AFS + MED	MED	Disposable Suture Device	T: L4/L5 = 7, L5/S1 = 15 C: L4/L5 = 10, L5/S1 = 18	VAS score	6 Month
Zhang (2019)	AFS + MED	MED	Disposable Suture Device (FAST-FIX)	T: L3/L4 = 5, L4/L5 = 20, L5/S1 = 14 C: L3/L4 = 6, L4/L5 = 22, L5/S1 = 16	Operative time Blood loss VAS score ODI score Disc height Recurrence	27.8 Month
He et al. (2018)	AFS + Discectomy	Discectomy	Disposable Suture Device (FAST-FIX)	T: L4/L5 = 12, L5/S1 = 9 C: L4/L5 = 14, L5/S1 = 10	Operative time Blood loss VAS score ODI score Recurrence	12 Month
Hu (2018)	AFS + Discectomy	Discectomy	Disposable Suture Device	T: L3/L4 = 4, L4/L5 = 20, L5/S1 = 15 C: L3/L4 = 3, L4/L5 = 21, L5/S1 = 13	Operative time Blood loss VAS score ODI score Recurrence	24 Month
Liu (2018)	AFS + MED	MED	Disposable Suture Device	NA	Operative time Blood loss Recurrence	18 Month
Song et al. (2018)	AFS + MED	PTED	Disposable Suture Device	T: L4/L5 = 13, L5/S1 = 7 C: L4/L5 = 10, L5/S1 = 6	Operative time Incision length Blood loss LOS VAS score JOA score ODI score	12.5 ± 1.82 Month
Xu et al. (2018)	AFS + MED	MED	Disposable Suture Device	NA	Blood loss VAS score ODI score Recurrence	NA
Guo et al. (2017)	AFS + OD	OD	Disposable Suture Device	NA	Operative time Blood loss LOS	6 Month
Jiang et al. (2017)	AFS + Discectomy	Discectomy	Disposable Suture Device	T: L3/L4 = 2, L4/L5 = 13, L5/S1 = 10 C: L3/L4 = 2, L4/L5 = 13, L5/S1 = 8	Operative time Incision length Blood loss VAS score ODI score Recurrence Complication	12 Month
Li et al. (2017)	AFS + OD	OD	Disposable Suture Device	T: L4/L5 = 18, L5/S1 = 14 C: L4/L5 = 17, L5/S1 = 15	Operative time Incision length Blood loss LOS VAS score ODI score Complication	18 Month
Lv (2017)	AFS + Discectomy	Discectomy	Disposable Suture Device	NA	Operative time Blood loss LOS VAS score ODI score	12 Month
Yu et al. (2017)	AFS + MED	MED	Disposable Suture Device	T: L4/L5 = 14, L5/S1 = 16 C: L4/L5 = 13, L5/S1 = 17	Operative time Blood loss VAS score JOA score ODI score	6 Month
Zhang et al. (2017a)	AFS + Discectomy	Discectomy	Disposable Suture Device	T: L4/L5 = 18, L5/S1 = 12 C: L4/L5 = 15, L5/S1 = 13	Operative time Incision length Blood loss LOS VAS score ODI score Recurrence	14.5 ± 1.3 Month
Zhang et al. (2017b)	AFS + OD	OD	Disposable Suture Device	T: L4/L5 = 23, L5/S1 = 16 C: L4/L5 = 33, L5/S1 = 14	Operative time Incision length Blood loss LOS Recurrence Complication	6 Month
Zhang (2017)	AFS + MED	MED	Disposable Suture Device	T: L4/L5 = 21, L5/S1 = 15 C: L4/L5 = 18, L5/S1 = 15	Operative time Blood loss VAS score ODI score Recurrence	18–32 Month
Zhu et al. (2017a)	AFS + OD	OD	Disposable Suture Device	L3/L4 = 8, L4/L5 = 45, L5/S1 = 39	Operative time Blood loss JOA score Recurrence Complication	18 Month
Zhu et al. (2017b)	AFS + MED	MED	Disposable Suture Device (FAST-FIX)	T: L3/L4 = 4, L4/L5 = 63, L5/S1 = 51 C: L3/L4 = 6, L4/L5 = 89, L5/S1 = 77	Operative time Blood loss VAS score ODI score Disc height Recurrence	T: 26.2 ± 10.4 Month C: 30.4 ± 11.2 Month
Li et al. (2016b)	AFS + OD	OD	Disposable Suture Device	T: L4/L5 = 8, L5/S1 = 4 C: L4/L5 = 10, L5/S1 = 4	Operative time Incision length Blood loss LOS VAS score ODI score Recurrence	12 Month
Zhang et al. (2015)	AFS + Discectomy	Discectomy	Disposable Suture Device	NA	VAS score JOA score ODI score Disc height	12–20 Month
Li et al. (2014)	AFS + MED	MED	Disposable Suture Device (FAST-FIX)	T: L3/L4 = 2, L4/L5 = 27, L5/S1 = 22 C: L3/L4 = 7, L4/L5 = 92, L5/S1 = 69	Operative time Blood loss Recurrence	28 Month
Bailey et al. (2013)	AFS + Discectomy	Discectomy	Xclose	T: L2/L3 or L3/L4 = 29, L4/L5 = 193, L5/L6 or L6/S1 = 2, L5/S1 = 273 C: L2/L3 or L3/L4 = 19, L4/L5 = 98, L5/L6 or L6/S1 = 1, L5/S1 = 146	Recurrence Complication	2 Year
Cai et al. (2013)	AFS + Discectomy	Discectomy	Disposable Suture Device	T: L3/L4 = 4, L4/L5 = 10, L5/S1 = 8 C: L3/L4 = 8, L4/L5 = 18, L5/S1 = 9	JOA score Recurrence	2.5 Year

AFS, annulus fibrosus suture; AUSS, arthroscopic-assisted uni-portal spinal surgery; C, control group; JOA, japanese orthopedic association; LOS, length of stay; MED, microendoscopic discectomy; NA, not available; OD, open discectomy; ODI, oswestry disability index; PEID, percutaneous endoscopic interlaminar discectomy; PELD, percutaneous endoscopic lumbar diskectomy; PTED, percutaneous transforaminal endoscopic discectomy; T, treatment group; TELD, transforaminal endoscopic lumbar discectomy; VAS, visual analogue scale.

**TABLE 3:** Summary data and analyses.

Outcomes	Subgroups	Number of studies	Effect estimate (MD or SMD or RR)	Heterogeneity		*P*-value for pooled result	Group deference (*P*-value)
				*P* value	I^2^ (%)		
Operative time	Total	46	4.85 [2.79 to 6.92]	<0.00001	94	<0.00001	​
	RCT	12	1.22 [-3.79 to 6.24]	<0.00001	95	0.63	0.08
	Non-RCT	34	6.11 [4.02 to 8.21]	<0.00001	92	<0.00001	
Incision length	Total	13	0.00 [-0.03 to 0.04]	0.11	34	0.84	
Blood loss	Total	41	0.32 [-1.17 to 1.80]	<0.00001	93	0.67	
	RCT	11	−5.90 [-13.66 to 1.86]	<0.00001	97	0.14	0.05
	Non-RCT	30	1.95 [0.82 to 3.08]	<0.00001	83	0.0007	
LOS	Total	23	0.06 [-0.49 to 0.62]	<0.00001	96	0.82	
	RCT	4	−0.42 [-1.53 to 0.69]	<0.00001	89	0.46	0.37
	Non-RCT	19	0.17 [-0.49 to 0.62]	<0.00001	97	0.60	
VAS score	Total	47	−0.24 [-0.33 to −0.14]	<0.00001	93	<0.00001	
	RCT	9	−0.34 [-0.57 to −0.12]	<0.00001	95	0.003	0.31
	Non-RCT	38	−0.21 [-0.32 to −0.10]	<0.00001	92	0.0002	​
	1-month follow-up	12	0.04 [-0.09 to 0.18]	0.02	51	0.53	0.01
	3-month follow-up	33	−0.19 [-0.39 to 0.01]	<0.00001	95	0.07	
	6-month follow-up	22	−0.28 [-0.46 to −0.09]	<0.00001	94	0.003	
	12-month follow-up	26	−0.23 [-0.38 to −0.09]	<0.00001	91	0.002	
JOA score	Total	12	0.85 [-0.23 to 1.93]	<0.00001	98	0.12	
	RCT	1	0.17 [-1.41 to 1.75]	-	-	0.83	0.46
	Non-RCT	11	0.91 [-0.22 to 2.04]	<0.00001	99	0.12	​
	1-month follow-up	1	0.09 [-0.90 to 1.08]	-	-	0.86	0.28
	3-month follow-up	3	1.00 [-1.29 to 3.29]	0.001	85	0.39	
	6-month follow-up	5	1.45 [0.23 to 2.67]	<0.00001	99	0.02	
	12-month follow-up	7	1.33 [0.15 to 2.51]	<0.00001	99	0.03	
ODI score	Total	44	−0.94 [-2.37 to 0.49]	<0.00001	99	0.20	
	RCT	9	−1.21 [-2.66 to 0.23]	<0.00001	92	0.10	0.76
	Non-RCT	35	−0.85 [-2.63 to 0.93]	<0.00001	100	0.35	​
	1-month follow-up	10	−0.74 [-1.32 to −0.17]	0.59	0	0.01	0.28
	3-month follow-up	30	−1.22 [-1.92 to −0.52]	<0.00001	86	0.0006	
	6-month follow-up	20	−1.13 [-1.91 to −0.34]	<0.00001	88	0.005	
	12-month follow-up	26	−0.15 [-1.08 to 0.78]	<0.00001	96	0.75	
Disc height	Total	11	1.36 [0.73 to 2.00]	<0.00001	96	<0.0001	
	RCT	1	−0.41 [-0.77 to −0.05]	-	-	0.03	<0.00001
	Non-RCT	10	1.55 [0.89 to 2.20]	<0.00001	96	<0.00001	
Recurrence	Total	48	0.34 [0.27 to 0.42]	0.99	0	<0.00001	
Complication	Total	9	0.80 [0.61 to 1.06]	0.84	0	0.12	

### Risk of bias assessment

The risk of bias for each included RCTs (He et al., 2025; Hu and Chen, 2024; Tian et al., 2023; Li J. et al., 2021; Xiao, 2021; Chen, 2020; He et al., 2018; Liu, 2018; Jiang et al., 2017; Lv, 2017; Zhang L. et al., 2017; Zhang, 2017; Li et al., 2014; Bailey et al., 2013) was evaluated using the RoB-2 instrument, and the findings are presented in Supplementary Figure S1. Among these, five trials were judged to be at low overall risk of bias (He et al., 2025; Tian et al., 2023; Lv, 2017; Zhang L. et al., 2017; Zhang, 2017). Eight trials raised some concerns, primarily in the domains of bias due to deviations from intended interventions (lack of surgeon blinding) and bias in measurement of the outcome (lack of patient blinding for subjective outcomes) (Hu and Chen, 2024; Li J. et al., 2021; Xiao, 2021; Chen, 2020; He et al., 2018; Jiang et al., 2017; Li et al., 2014; Bailey et al., 2013). One study was judged to be at high risk due to concerns regarding the randomization process (Liu, 2018). The ROBINS-I tool was adopted to assess the quality of evidence of included non-RCTs (Gao and Liang, 2025; Zhang Q. et al., 2025; Zhou et al., 2025; Chen et al., 2024; Fu et al., 2024; He et al., 2024; Li and Du, 2024; Liu et al., 2024; Peng et al., 2024; Shi et al., 2024; Sun et al., 2024; Xi et al., 2024; Yu et al., 2024; Zhao Y. F. et al., 2024; Liang et al., 2023; Liu et al., 2023; Song et al., 2023; Wu et al., 2023; Ding et al., 2022; Tang and Xu, 2022; Wang Z. et al., 2022; Xu et al., 2022; Yao et al., 2022; Jiao et al., 2021; Zhang L. et al., 2021; Fu et al., 2020; Ren et al., 2020; Song et al., 2020; Chen et al., 2019; Li, 2019; Yi et al., 2019; Zhang, 2019; Hu, 2018; Song et al., 2018; Xu et al., 2018; Guo et al., 2017; Li et al., 2017; Yu et al., 2017; Zhang G. G. et al., 2017; Zhu G. H. et al., 2017; Zhu Z. Y. et al., 2017; Li J. et al., 2016; Zhang et al., 2015), as shown in Supplementary Figure S2. Thirteen studies were rated at low overall risk of bias (Chen et al., 2024; He et al., 2024; Sun et al., 2024; Xi et al., 2024; Yu et al., 2024; Zhao Y. F. et al., 2024; Wu et al., 2023; Ding et al., 2022; Zhang L. et al., 2021; Li, 2019; Hu, 2018; Guo et al., 2017; Zhu Z. Y. et al., 2017). The twenty-eight studies were rated at moderate risk (Gao and Liang, 2025; Zhang Q. et al., 2025; Zhou et al., 2025; Fu et al., 2024; Li and Du, 2024; Liu et al., 2024; Peng et al., 2024; Shi et al., 2024; Liang et al., 2023; Liu et al., 2023; Song et al., 2023; Tang and Xu, 2022; Wang Z. et al., 2022; Jiao et al., 2021; Fu et al., 2020; Song et al., 2020; Chen et al., 2019; Yi et al., 2019; Zhang, 2019; Song et al., 2018; Xu et al., 2018; Li et al., 2017; Yu et al., 2017; Zhang G. G. et al., 2017; Zhu G. H. et al., 2017; Li J. et al., 2016; Zhang et al., 2015; Cai et al., 2013). The most prevalent sources of potential bias in these studies were bias due to confounding (differences in surgeon experience), bias in the selection of participants (associated with the retrospective cohort design), and selective reporting of outcomes. Three studies were rated at serious risk of bias, mainly due to issues such as significant deviations from the intended intervention or substantial loss to follow-up (Xu et al., 2022; Yao et al., 2022; Ren et al., 2020).

### Meta-analysis results

#### Operative time

A total of 46 studies with 4,413 participants reported operative time in the study. There was significant heterogeneity across studies (*P* < 0.00001, *I* ^*2*^ = 94%). This result indicates that statistically significant differences in operative time exist between the two groups (MD = 4.85, 95% CI: 2.79 to 6.92, *P* < 0.00001) (Supplementary Figure S3).

Further subgroup analyses were conducted based on variations in study design to explore the influence of these variables on the therapeutic effect. These analyses revealed that the overall findings were robust, although a minority of subgroups did not show significant benefits. Results revealed no significant impact on operative time in subgroups of RCT studies (MD = 1.22, 95% CI: −3.79 to 6.24, *P* = 0.63) (Table 3; Supplementary Figure S4).

#### Incision length

A total of 13 studies with 831 participants reported incision length in the study. There was low heterogeneity across studies (*P* = 0.11, *I* ^*2*^ = 34%). This result indicates that there are no statistically significant differences in incision length between the two groups (MD = 0.00, 95% CI: −0.03 to 0.04, *P* = 0.84) (Supplementary Figure S5).

#### Blood loss

A total of 41 studies with 3,913 participants reported blood loss in the study. There was significant heterogeneity across studies (*P* < 0.00001, *I* ^*2*^ = 93%). This result indicates that there are no statistically significant differences in blood loss between the two groups (MD = 0.32, 95% CI: −1.17 to 1.80, *P* = 0.67) (Supplementary Figure S6).

Subgroup analyses were conducted according to different study designs. Pooled analysis showed that the significant impact on blood loss in subgroups of Non-RCT studies (MD = 1.95, 95% CI: 0.82 to 3.08, *P* = 0.0007) (Table 3; Supplementary Figure S7).

#### LOS

A total of 23 studies with 1824 participants reported LOS in the study. There was significant heterogeneity across studies (*P* < 0.00001, *I* ^*2*^ = 96%). This result indicates that there are no statistically significant differences in LOS between the two groups (MD = 0.06, 95% CI: −0.49 to 0.62, *P* = 0.82) (Supplementary Figure S8).

Further subgroup analysis was conducted. The results were consistent across all subgroups, confirming that these variables had no significant effect on reducing the LOS (Table 3; Supplementary Figure S9).

#### VAS score

A total of 47 studies with 4,090 participants reported the VAS score in the study. There was significant heterogeneity across studies (*P* < 0.00001, *I* ^*2*^ = 93%). This result indicates that statistically significant differences in VAS score between the two groups (MD = −0.24, 95% CI: −0.33 to −0.14, *P* < 0.00001) (Supplementary Figure S10).

Subgroup analyses were conducted according to different study designs and lengths of follow-up. These findings indicate that the overall results remain robust, despite the lack of statistically significant effects in a minority of subgroups. When stratified according to study design, the subgroup analyses yielded results consistent with the overall estimate, also demonstrating statistical significance. However, in the subgroup analysis stratified by follow-up time, no statistically significant differences in VAS score between the two groups were observed at the 1-month (MD = 0.04, 95% CI: −0.09 to 0.18, *P* = 0.53) and 3-month postoperative (MD = −0.19, 95% CI: −0.39 to 0.01, *P* = 0.07) follow-ups (Table 3; Supplementary Figures S11-S12).

#### JOA score

A total of 12 studies with 1,279 participants reported the JOA score in the study. There was significant heterogeneity across studies (*P* < 0.00001, *I* ^*2*^ = 98%). This result indicates that there are no statistically significant differences in JOA score between the two groups (MD = 0.85, 95% CI: −0.23 to 1.93, *P* = 0.12) (Supplementary Figure S13).

Subgroup analysis based on study design demonstrated results consistent with the overall estimate, without statistical significance for either. Conversely, a statistically significant difference between the two groups emerged at the 6-month and 12-month postoperative follow-ups. In contrast, the analysis demonstrated statistically significant differences in JOA scores between the groups at the 6-month (MD = 1.45, 95% CI: 0.23 to 2.67, *P* = 0.02) and 12-month postoperative (MD = 1.33, 95% CI: 0.15 to 2.51, *P* = 0.03) assessments (Table 3; Supplementary Figures S14-S15).

#### ODI score

A total of 44 studies with 3,770 participants reported the ODI score in the study. There was significant heterogeneity across studies (*P* < 0.00001, *I* ^*2*^ = 99%). This result indicates that there are no statistically significant differences in ODI score between the two groups (MD = −0.94, 95% CI: −2.37 to 0.49, *P* = 0.20) (Supplementary Figure S16).

Subgroup analysis based on study design demonstrated results consistent with the overall estimate, without statistical significance for either. Conversely, a statistically significant difference between the two groups emerged at the 1-month, 6-month, and 12-month postoperative follow-ups. In contrast, the analysis demonstrated statistically significant differences in ODI scores between the groups at the 1-month (MD = −0.74, 95% CI: −1.32 to −0.17, *P* = 0.01), 3-month (MD = −1.22, 95% CI: −1.92 to −0.52, *P* = 0.0006) and 6-month postoperative (MD = −1.13, 95% CI: −1.91 to −0.34, *P* = 0.005) assessments (Table 3; Supplementary Figures S17-S18).

#### Disc height

A total of 11 studies with 1,176 participants reported disc height in the study. There was significant heterogeneity across studies (*P* < 0.00001, *I* ^*2*^ = 96%). This result indicates that statistically significant differences in disc height exist between the two groups (SMD = 1.36, 95% CI: 0.73 to 2.00, *P* < 0.0001) (Supplementary Figure S19).

Additional stratified analyses revealed that the effect estimates for all subgroups remained congruent with the overall result (Table 3; Supplementary Figure S20).

#### Recurrence

A total of 48 studies with 4,999 participants reported recurrence in the study. There was low significant heterogeneity across studies (*P* = 0.99, *I* ^*2*^ = 0%). This result indicates that statistically significant differences in recurrence exist between the two groups (RR = 0.34, 95% CI: 0.27 to 0.42, *P* < 0.00001) (Supplementary Figure S21).

#### Complication

A total of nine studies with 1,466 participants reported complication in the study. There was low significant heterogeneity across studies (*P* = 0.84, *I* ^*2*^ = 0%). This result indicates that no statistically significant differences in complication exist between the two groups (RR = 0.80, 95% CI: 0.61 to 1.06, *P* = 0.12) (Supplementary Figure S22).

#### Sensitivity analysis

To verify the reliability of the results in this study, sensitivity analyses were conducted on various outcome indicators, including operative time, blood loss, LOS, VAS score, JOA score, ODI score, and disc height. For blood loss, the effect size shifted following the exclusion of Tian’s study (Tian et al., 2023) (*I* ^*2*^ = 79%, *P* = 0.002), indicating it may be the source of heterogeneity. For the JOA score, the effect size changed after excluding Zhou’s study (Zhou et al., 2025) (*I* ^*2*^ = 98%, *P* = 0.04) and Jiao’s study (Jiao et al., 2021) (*I* ^*2*^ = 98%, *P* = 0.0007). Similarly, for the JOA score, the results became significant after excluding the adolescent population studies (Jiao et al., 2021; Song et al., 2018). Sensitivity analyses of other outcomes confirmed the stability of pooled effect estimates. Complete sensitivity analysis data are provided in Supplementary Tables S1-S7.

#### Publication bias

For the outcomes of operative time, incision length, blood loss, LOS, VAS score, JOA score, ODI score, disc height, and recurrence, asymmetry was observed in the funnel plots. Therefore, we performed an Egger’s test to confirm the risk of publication bias. However, Egger’s test did not detect significant bias for operative time, incision length, blood loss, LOS, VAS score, JOA score, and ODI score, suggesting stable effect estimates for these outcomes. For disc height and recurrence, Egger’s test suggested potential bias. Therefore, the Trim and Fill method was used for correction. The effect size after disc height correction was (SMD = 2.04, 95% CI: 1.01 to 4.09, *P* = 0.046), and the effect size after recurrence correction was (RR = 0.52, 95% CI: 0.42 to 0.66, *P* < 0.001). Detailed results are provided in Supplementary Figures S23-S31 and Supplementary Table S8.

#### GRADE evaluation

Based on the principles of the GRADE evaluation, we assessed the quality of the evidence in terms of operative time, incision length, blood loss, LOS, VAS score, JOA score, ODI score, disc height, recurrence, and complication. According to Supplementary Table S9, the evidence for the incision length was classified as moderate quality, JOA score and complication were classified as low quality, while the other evidence was of very low quality.

## Discussion

LDH is a common clinical spinal disorder. With societal acceleration and evolving lifestyles, its prevalence has been steadily increasing (Zhang A. S. et al., 2023; Xu et al., 2023; Zhang S. et al., 2025). The surgical management of LDH is undergoing continuous evolution, driven by advances in mezdical technology. Nevertheless, achieving better efficacy, improved patient prognosis, and reducing postoperative recurrence rates persists as key objectives in clinical practice (Bhandutia et al., 2025; Liu et al., 2025). Some studies have found that although discectomy relieves nerve root compression, it can exacerbate the annulus fibrosus defect, thereby potentially increasing the risk of symptom recurrence and reoperation (Miller et al., 2018; Lorio et al., 2020; Babington et al., 2025). Therefore, intraoperative restoration of annulus fibrosus integrity is of critical importance. The annular closure device typically consists of a woven polymer mesh that is anchored into the adjacent vertebral bodies with titanium bone anchors (Tavakoli et al., 2020). Following discectomy, the device is placed directly into the annulus fibrosus defect. This helps preserve residual nucleus pulposus, maintain disc height, and promote natural healing, thereby slowing degenerative progression (Kurzbuch et al., 2022; Jung et al., 2023). Concurrently, it restores disc integrity of the disc space and reduces the risk of recurrent herniation (Lorio et al., 2020; Kienzler et al., 2021a). However, we cannot ignore the potential issues of device migration, implantation failure, and related complications (Kienzler et al., 2021b; McClure et al., 2024; Jin et al., 2025b). An alternative approach involves the direct suturing of the damaged annulus fibrosus during surgery, but has yielded inconsistent clinical efficacy (Gao and Liang, 2025; He et al., 2025; Zhang Q. et al., 2025). Accordingly, the present study was conducted to assess the clinical value of the AFS technique in the surgical management of LDH.

In this meta-analysis, 58 studies (Gao and Liang, 2025; He et al., 2025; Zhang Q. et al., 2025; Zhou et al., 2025; Chen et al., 2024; Fu et al., 2024; He et al., 2024; Hu and Chen, 2024; Li and Du, 2024; Liu et al., 2024; Peng et al., 2024; Shi et al., 2024; Sun et al., 2024; Xi et al., 2024; Yu et al., 2024; Zhao Y. F. et al., 2024; Liang et al., 2023; Liu et al., 2023; Song et al., 2023; Tian et al., 2023; Wu et al., 2023; Ding et al., 2022; Tang and Xu, 2022; Wang Z. et al., 2022; Xu et al., 2022; Yao et al., 2022; Jiao et al., 2021; Li J. et al., 2021; Xiao, 2021; Zhang L. et al., 2021; Chen, 2020; Fu et al., 2020; Ren et al., 2020; Song et al., 2020; Chen et al., 2019; Li, 2019; Yi et al., 2019; Zhang, 2019; He et al., 2018; Hu, 2018; Liu, 2018; Song et al., 2018; Xu et al., 2018) were enrolled, which included 5,765 patients with LDH. Our analysis demonstrates significant differences in operative time, VAS score, disc height, and recurrence between the surgery with AFS and the conventional surgery. However, no significant differences were observed between approaches in the incision length, blood loss, LOS, JOA score, ODI score, and complication.

The study results demonstrated that the AFS group had a longer operative time than the control group, with a statistically significant difference. In the AFS group, after removing the herniated nucleus pulposus, the ruptured annulus fibrosus was cleaned and sutured using a suitable method. However, the control group did not receive any treatment for the ruptured annulus fibrosus. Though the surgeries for both groups were performed by the same experienced surgeon, the AFS technique is still emerging and can be a challenge for surgeons, contributing to the longer operative time (Song et al., 2023; Yi et al., 2019). It’s anticipated that as the AFS technique advances and becomes more widespread, the operative time gap between the groups will decrease. Concerning operative time, the heterogeneity observed may be attributed to the fact that some research centers have limited experience with the AFS technique and are still on a slow learning curve. Additionally, most studies do not clarify whether anesthesia administration and patient positioning times are included in the operative time.

In this systematic review and meta-analysis, we found no significant differences between the two groups in terms of incision length, blood loss, and LOS. A single-center RCT involving 80 patients with LDH indicated similar incision lengths for percutaneous transforaminal endoscopic discectomy combined with AFS and percutaneous transforaminal endoscopic discectomy alone (Hu and Chen, 2024). Similarly, Liu’s study showed that the endoscopic AFS technique does not cause extra patient injury (Liang et al., 2023; Liu et al., 2023).

Greater blood loss in LDH surgery is linked to higher perioperative complication risks. Thus, blood loss is an important outcome indicator of surgical safety and quality. Some scholars think that, as both surgeries are done endoscopically, the AFS technique does not require more removal of the facet joint and ligamentum flavum, so it will not cause extra trauma (Liu et al., 2023). Similarly, compared with the control group, the AFS group does not increase blood loss (He et al., 2025). This may be because the transforaminal endoscopic technique allows timely hemostasis and provides a clear surgical field, creating good conditions for AFS and effectively avoiding medical injury in the operation process. Moreover, the annulus fibrosus has collagen fibers in concentric circles and lacks a significant blood supply.

The lack of difference in LOS may be due to the similar impacts of the two techniques on postoperative recovery and overall health, leading to comparable hospitalization times. A relevant RCT has compared percutaneous transforaminal endoscopic discectomy and percutaneous transforaminal endoscopic discectomy combined with AFS in elderly patients with LDH and found no significant difference in LOS. This indicates that AFS adjuvant therapy does not bring additional risks or require a longer recovery time. It’s important to note that there are differences in hospitalization management policies across hospitals.

Lumbar pain is a primary symptom of patients with LDH, so observing pain changes in patients is crucial. The VAS score, known for its simplicity and patient acceptance, is widely used in clinical research. Studies indicate that surgery combined with AFS can effectively alleviate pain symptoms in patients. Chen’s study found that this combined approach significantly reduces both the lumbar VAS score and leg VAS score (Chen, 2020). Similarly, Yao’s study observed effective pain relief after performing interrupted sutures on the ruptured annulus fibrosus following nucleotomy (Yao et al., 2022). However, some studies have not found AFS to be significantly advantageous in postoperative pain improvement (Song et al., 2020). LDH occurs when the annulus fibrosus ruptures due to various reasons, allowing the nucleus pulposus to directly compress or stretch the nerve root (Zhang H. et al., 2025; Zhou M. et al., 2023). The nucleus pulposus contains a variety of inflammatory mediators like PLA2, histamine, lactate, substance P, and others. When these mediators come into contact with nerve roots due to herniation, they trigger the release of other inflammatory factors, causing persistent pain (Yuan et al., 2023; Zhang S. et al., 2021). Both treatments relieve pain by removing the herniated nucleus pulposus to decompress nerve roots, so there is no difference in the short-term efficacy observation. Xu’s research compared the early pain-relieving effects of AFS combined with nucleotomy and nucleotomy alone in 65 participants and found them similar (Xu et al., 2022). Our study also found no significant differences in early postoperative VAS score between the two groups, possibly due to their similar main objectives. Zhu and his team found that many patients still experience pain after surgery, likely due to residual nucleus pulposus debris irritating nerve roots (Zhu et al., 2016). The nucleus pulposus, as an immune-privileged tissue that is usually isolated from systemic circulation, can trigger autoimmune responses when exposed to the immune system through herniation. Intraoperative application of AFS as an adjunctive procedure enables immediate closure of annular defects, thereby accelerating the repair process at the rupture site. Meanwhile, this technique closes the channel between the nucleus pulposus tissue and the spinal canal, effectively blocking the stimulation of nerve roots by residual nucleus pulposus fragments. This creates a relatively enclosed internal environment within the intervertebral disc, reducing the release of inflammatory factors and minimizing chemical irritation to nerve roots, thereby relieving postoperative pain. Without intraoperative repair of the annulus fibrosus, its rupture persists with limited self-healing ability, requiring a prolonged fibrosus scar to achieve this. During this period, residual nucleus pulposus remains exposed to the immune system, where systemic circulation recognizes it as a foreign antigen, triggering immune attacks (Ye et al., 2022). Some researchers suggest that persistent inflammatory mediator stimulation at unrepaired annulus fibrosus defects contributes to chronic postoperative pain (Carragee et al., 2003). An RCT demonstrated that adjunctive AFS can reduce serum levels of β-endorphin, prostaglandin E2, and 5-hydroxytryptamine (Li, 2024). Additionally, Chen’s study compared concentrations of TNF-α and phospholipase A2 in serum and drainage fluid before and after surgery with versus without AFS, finding that combined AFS significantly reduces inflammatory factor release (Chen et al., 2019).

The JOA score comprehensively evaluates the clinical improvement of patients across four domains, including subjective symptoms, clinical signs, daily living ability, and bladder function. In Tang’s study, both groups showed increased postoperative JOA scores, yet the AFS group had a higher score (Tang and Xu, 2022). This implies that AFS positively enhances lumbar functional recovery, physical performance, and quality of life. Similarly, a single-center retrospective study of 92 patients demonstrated higher JOA scores in those receiving adjunctive AFS compared to controls (Zhu G. H. et al., 2017). This may be attributed to AFS restoring postoperative integrity of the annulus fibrosus, which facilitates early postoperative mobilization and functional exercise, thereby shortening the recovery period.

The ODI is clinically used to assess the impact of low back pain on daily living. Both patient groups showed similar improvements in ODI. An RCT revealed that patients receiving adjunctive AFS improved ODI at 6, 12, and 18 months postoperatively (Lv, 2017). Hu’s study found that both surgeries, combined with AFS and surgery alone, improved ODI in patients, with no significant difference between groups 1 year post-operation (Hu and Chen, 2024). This aligns with a meta-analysis comparing discectomy with annulus fibrosus repair versus discectomy alone in LDH patients, which found no significant difference in ODI improvement between techniques (Wang et al., 2024). This meta-analysis reinforces our observation that AFS is a safe adjunctive approach, yielding comparable clinical ODI improvement, though its efficacy requires further validation through large-scale studies.

Our study revealed significant differences in postoperative disc height between the surgery combined with AFS and the surgery group, consistent with multiple studies (Chen, 2020; Fu et al., 2020). Li and his team analyzed 285 single-level LDH patients treated with percutaneous transforaminal endoscopic discectomy, finding that a severe degree of disc degeneration, smaller disc height index, and larger postoperative annulus fibrosus defects may be associated with early recurrence (Li et al., 2022). Scholars have suggested that AFS alone may not provide significant additional benefits for postoperative pain and functional recovery, but could help maintain disc height (Xu et al., 2022). The annulus fibrosus comprises inner and outer layers. The inner layer bears hydrostatic pressure from the nucleus pulposus, while the outer layer restricts excessive spinal motion and withstands tensile forces (Zhu et al., 2024; Li C. et al., 2021). Ren’s study demonstrated that circumferential suturing of annulus fibrosus defects during microendoscopic discectomy preserves disc height (Ren et al., 2020). Maintaining disc height supports spinal stability and reduces adjacent segment degeneration. Fu’s research also reported that surgery combined with AFS effectively improves disc height and mobility in young patients, promoting early functional recovery (Fu et al., 2020). A healthy annulus fibrosus exhibits strong contractility to cushion spinal loads and restore disc shape, playing a vital role in spinal mechanics (Castro and Gonçalves, 2025). An animal study found that annulus fibrosus repair limits motion in surgical segments, enhancing spinal stability (Chen et al., 2023). Similarly, Wang’s team observed in a goat model that AFS preserves disc height and delays disc degeneration (Wang et al., 2018).

One of the key findings of our investigation is the reduced recurrence in patients undergoing AFS. Primary surgery may alter local anatomy, induce epidural fibrosis, and promote scar tissue formation, all of which increase the technical difficulty and risks of revision surgery. Additionally, these factors impose physical and psychological burdens on patients. Studies have identified age, Pfirrmann grade, and rehabilitation duration as factors influencing recurrence after percutaneous transforaminal endoscopic discectomy (Zhu L. et al., 2025). Zhao et al. further highlighted age, disease duration, Pfirrmann grade, annulus fibrosus defects, and incomplete nucleus pulposus removal as recurrence risk factors, corroborated by other scholars (Zhao Y. G. et al., 2024; Zhong et al., 2024). Yao et al. reported 35 cases with no recurrence and reoperations over a 2-year follow-up after nucleotomy combined with AFS, with imaging showing no accelerated disc degeneration (Yao et al., 2022). Ding’s study similarly demonstrated that AFS after nucleotomy significantly reduces recurrence risk and reoperation demand (Ding et al., 2022). The extent of nucleus pulposus removal during surgery is critical for ensuring therapeutic efficacy and preventing recurrence. Excessive intraoperative removal risks disc height loss and accelerated disc degeneration, potentially compromising spinal stability. Conversely, limited nucleus pulposus removal without annulus fibrosus repair may lead to postoperative recurrence in some patients due to residual nucleus pulposus tissue, inflammatory mediators, and torn annular fragments. AFS technique restores annulus fibrosus mechanical integrity, facilitating postoperative annulus fibrosus healing and maintaining intervertebral disc function. On the one hand, it relieves nerve root compression while preserving healthy nucleus pulposus to delay degeneration. On the other hand, it prevents postoperative physical and chemical irritation of nerve roots, thereby minimizing recurrence. Finally, no significant difference in complication between the two surgical approaches. As both surgeries involve careful spinal structure management, they consequently yield comparable safety results. This indicates that adjunctive AFS does not cause additional harm, supporting its potential as a safe supplement to LDH surgery.

Furthermore, significant publication bias was detected for the two significant outcomes of disc height and recurrence. This phenomenon may be attributed to the tendency of smaller studies to report exaggerated effect sizes, leading to their preferential publication, alongside methodological heterogeneities such as variations in assessment timing. The statistical significance of these findings persisted after trim-and-fill correction. Nevertheless, caution is advised in interpreting these outcomes due to the initial presence of bias.

### Strengths and limitations

To the best of our knowledge, this is the first meta-analysis and systematic review evaluating the efficacy and safety of surgery combined with AFS for LDH from operative time, incision length, blood loss, LOS, VAS score, JOA score, ODI score, disc height, recurrence, and complication. Despite significant findings, our study has several limitations. First, most of the studies included were non-randomized, which may introduce bias and confounding factors. There are some differences in RCTs and non-RCTs’ results that should be kept in mind while interpreting these results. Second, some outcomes are based on scale scores, which may be subject to varying interpretations by researchers and patients, potentially influencing the results. These study results should be interpreted with caution. Third, there was a high level of heterogeneity in some outcomes, such as operative time, which could be due to patient populations, surgical protocols, annulus fibrosus defect severity, extent of nucleus removal, and follow-up duration. Future studies could employ meta-regression analysis to further explore the sources of heterogeneity. Fourth, while all studies utilized AFS, variations in incision direction, incision length, suturing methods, and stitch spacing might impact findings. Meanwhile, as all included studies were conducted in China, the generalizability of the findings to other populations and healthcare contexts may be limited. To enhance the universality of the findings, it is recommended that verification studies be carried out in multiple regions in the future. Fifth, surgical outcomes depend on the surgeon’s technical skill and experience, which vary across studies and introduce bias. Sixth, the overall certainty of evidence for most outcomes, as evaluated by the GRADE framework, was rated as low or very low. These ratings were consistently downgraded due to serious concerns regarding study design (inclusion of multiple non-randomized designs), risk of bias, and inconsistency (substantial unexplained statistical heterogeneity). Therefore, further high-quality RCTs are necessary to confirm our findings.

### Prospects for future research

Future studies should optimize experimental protocols in the following aspects. First, current research lacks data on the long-term efficacy and potential complications of AFS. Future studies should use rigorous control groups, randomization, and high-quality blinding to enhance the reliability. Second, during surgery, surgeons should minimize puncture attempts and employ gentle, gradual knot tying to avoid secondary annular damage. Excessive punctures may compromise annular integrity, impairing surgical outcomes. Current physical suturing methods require refinement to enhance closure effectiveness. Finally, while AFS techniques focus on restoring mechanical annulus fibrosus integrity, future research should integrate cell therapy, gene therapy, and tissue engineering to restore the annulus fibrosus’s physiological function.

## Conclusion

In conclusion, current evidence suggests that AFS may offer potential benefits in LDH surgery. However, given the overall low quality of the included studies, these findings should be interpreted with caution. More large-scale, multicenter RCTs are needed in the future to confirm the clinical efficacy and safety of AFS as an adjuvant therapy for LDH.

## Data Availability

The original contributions presented in the study are included in the article/Supplementary Material, further inquiries can be directed to the corresponding authors.
