# Supplementary material for: The efficacy and safety of annulus fibrosus suture as adjuvant therapy for lumbar disc herniation: a systematic review and meta-analysis
Source: Front Bioeng Biotechnol. 2026 Jan 27;13:1741738. doi: 10.3389/fbioe.2025.1741738 (PMC12886413; doi:10.3389/fbioe.2025.1741738)
Supplement: Supplementary file 1 [file Supplementaryfile1.docx]

**Supplementary Material**

The efficacy and safety of annulus fibrosus suture as adjuvant therapy for lumbar disc herniation: a systematic review and meta-analysis

Search strategies 4

Figure S1. Assessment of risk of bias of randomized trials with RoB-2. 7

Figure S2. Assessment of risk of bias of non-randomized trials with ROBINS-I. 8

Figure S3. Forest plot for operative time. 9

Figure S4. Subgroup analysis of operative time by study design. 10

Figure S5. Forest plot for incision length. 11

Figure S6. Forest plot for blood loss. 11

Figure S7. Subgroup analysis of blood loss by study design. 12

Figure S8. Forest plot for LOS. 13

Figure S9. Subgroup analysis of LOS by study design. 13

Figure S10. Forest plot of VAS score. 14

Figure S11. Subgroup analysis of VAS score by study design. 15

Figure S12. Subgroup analysis of VAS score by follow-up duration. 16

Figure S13. Forest plot of JOA score. 17

Figure S14. Subgroup analysis of JOA score by study design. 17

Figure S15. Subgroup analysis of JOA score by follow-up duration. 18

Figure S16. Forest plot of ODI score. 19

Figure S17. Subgroup analysis of ODI score by study design. 20

Figure S18. Subgroup analysis of ODI score by follow-up duration. 21

Figure S19. Forest plot for disc height. 21

Figure S20. Subgroup analysis of disc height by study design. 22

Figure S21. Forest plot for recurrence. 23

Figure S22. Forest plot for complication. 23

Table S1. Seneitivity analysis for operative time. 24

Table S2. Seneitivity analysis for blood loss. 28

Table S3. Seneitivity analysis for LOS. 31

Table S4. Seneitivity analysis for VAS score. 33

Table S5. Seneitivity analysis for JOA score. 37

Table S6. Seneitivity analysis for ODI score. 38

Table S7. Seneitivity analysis for disc height. 42

Figure S23. The funnel plot of operative time. 43

Figure S24. The funnel plot of incision length. 43

Figure S25. The funnel plot of blood loss. 44

Figure S26. The funnel plot of LOS. 44

Figure S27. The funnel plot of VAS score. 45

Figure S28. The funnel plot of JOA score. 45

Figure S29. The funnel plot of ODI score. 46

Figure S30. The funnel plot of disc height. 46

Figure S31. The funnel plot of recurrence. 47

Table S8. Publication bias of the included studies. 47

Table S9. GRADE evaluation of evidence quality. 48

**Search strategies**

***PubMed:***

#1: “annulus fibrosus repair” [Title/Abstract] OR “annulus fibrosus” [Title/Abstract] OR “annulus fibrosus suture” [Title/Abstract] OR “annular repair” [Title/Abstract] OR “annulus repair” [Title/Abstract] OR “anular suture” [Title/Abstract] OR “anulus repair” [Title/Abstract] OR “annular suture” [Title/Abstract] OR “annuloplasty” [Title/Abstract]

#2: “lumbar disc herniation” [Title/Abstract] OR “lumbar disc protrusion” [Title/Abstract] OR “intervertebral disc displacement” [Title/Abstract] OR “lumbar intervertebral disc herniation” [Title/Abstract] OR “lumbar” [Title/Abstract] OR “hernia disci lumbalis” [Title/Abstract] OR “LDH” [Title/Abstract]

#3: #1 AND #2

***Web of Science:***

#1: TS=(“annulus fibrosus repair” OR “annulus fibrosus” OR “annulus fibrosus suture” OR “annular repair” OR “annulus repair” OR “anular repair” OR “anulus repair” OR “annular suture” OR “annuloplasty”)

#2: TS=(“lumbar disc herniation” OR “lumbar disc protrusion” OR “intervertebral disc displacement” OR “lumbar intervertebral disc herniation” OR “lumbar” OR “hernia disci lumbalis” OR “LDH”)

#3: #1 AND #2

***EMBASE:***

#1: 'annulus fibrosus repair':ab,ti OR 'annulus fibrosus':ab,ti OR 'annulus fibrosus suture':ab,ti OR 'annular repair':ab,ti OR 'annulus repair':ab,ti OR 'anular repair':ab,ti OR 'anulus repair':ab,ti OR 'annular suture':ab,ti OR 'annuloplasty':ab,ti

#2: 'lumbar disc herniation':ab,ti OR 'lumbar disc protrusion':ab,ti OR 'intervertebral disc displacement':ab,ti OR 'lumbar intervertebral disc herniation':ab,ti OR 'lumbar':ab,ti OR 'hernia disci lumbalis':ab,ti OR 'LDH':ab,ti

#3: #1 AND #2

***Cochrane Library：***

#1: 'annulus fibrosus repair':ti,ab,kw OR 'annulus fibrosus':ti,ab,kw OR 'annulus fibrosus suture':ti,ab,kw OR 'annular repair':ti,ab,kw OR 'annulus repair':ti,ab,kw OR 'anular repair':ti,ab,kw OR 'anulus repair':ti,ab,kw OR 'annular suture':ti,ab,kw OR 'annuloplasty':ti,ab,kw

#2: 'lumbar disc herniation':ti,ab,kw OR 'lumbar disc protrusion':ti,ab,kw OR 'intervertebral disc displacement':ti,ab,kw OR 'lumbar intervertebral disc herniation':ti,ab,kw OR 'lumbar':ti,ab,kw OR 'hernia disci lumbalis':ti,ab,kw OR 'LDH':ti,ab,kw

#3: #1 AND #2

***Chinese National Knowledge Infrastructure：***

#1: SU=纤维环 + 纤维环缝合 + 纤维环修复

#2: SU=腰椎间盘突出症 + 腰椎间盘突出 + 腰痛 + 腰痹 + LDH

#3: #1 AND #2

***Chinese Science and Technology Journal database：***

#1: 题名或关键词：纤维环 + 纤维环缝合 + 纤维环修复

#2: 题名或关键词：腰椎间盘突出症 + 腰椎间盘突出 + 腰痛 + 腰痹 + LDH

#3: #1 AND #2

***WanFang database：***

#1: 题名或关键词：纤维环 or 纤维环缝合 or 纤维环修复

#2: 题名或关键词：腰椎间盘突出症 or 腰椎间盘突出 or 腰痛 or 腰痹 or LDH

#3: #1 AND #2

***Chinese Biological Literature database：***

#1: 常用字段：纤维环 OR 纤维环缝合 OR 纤维环修复

#2: 常用字段：腰椎间盘突出症 OR 腰椎间盘突出 OR 腰痛 OR 腰痹 OR LDH

#3: #1 AND #2


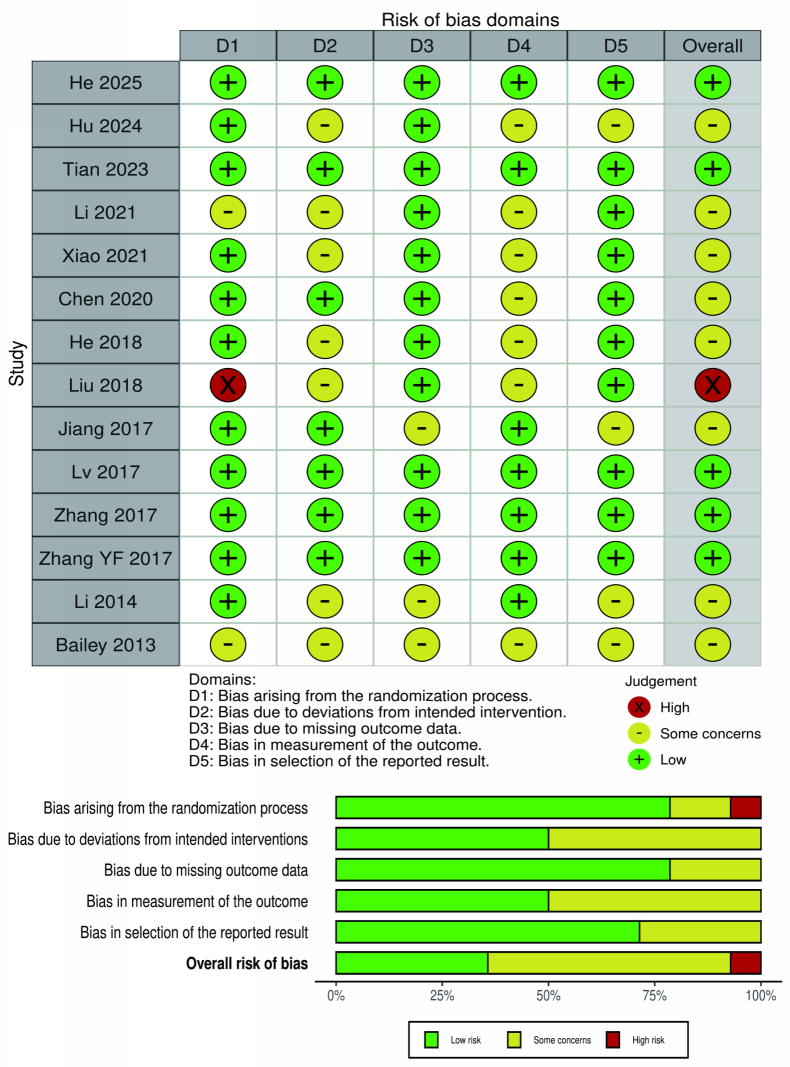


**Figure S1.** Assessment of risk of bias of randomized trials with RoB-2.


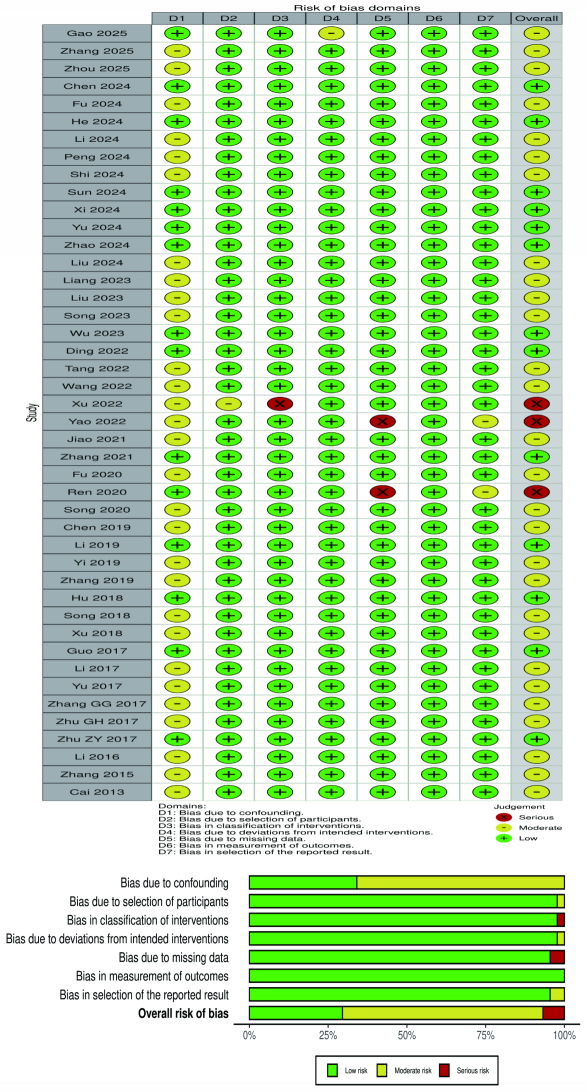


**Figure S2.** Assessment of risk of bias of non-randomized trials with ROBINS-I.


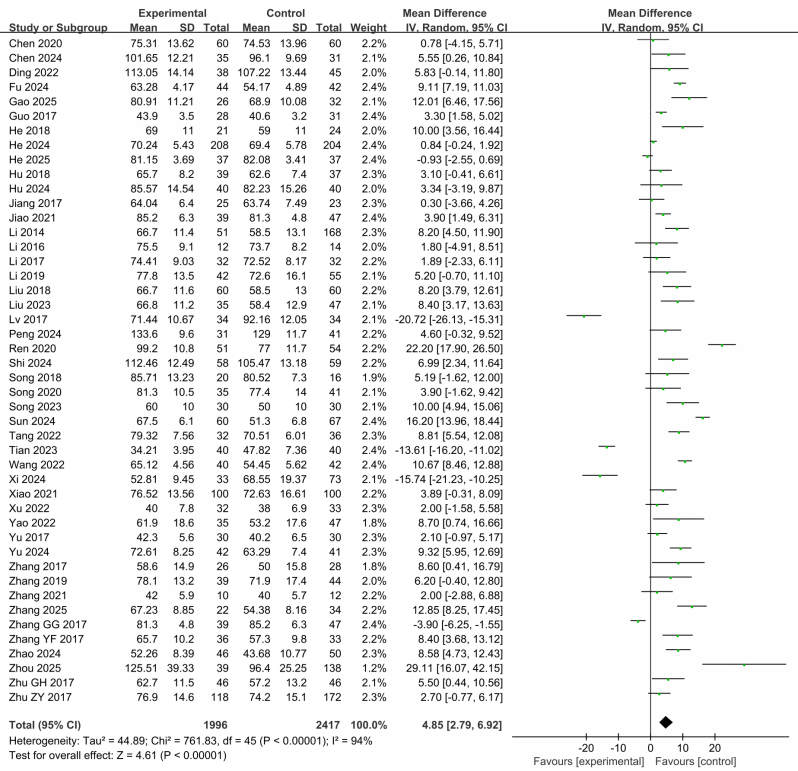


**Figure S3.** Forest plot for operative time.


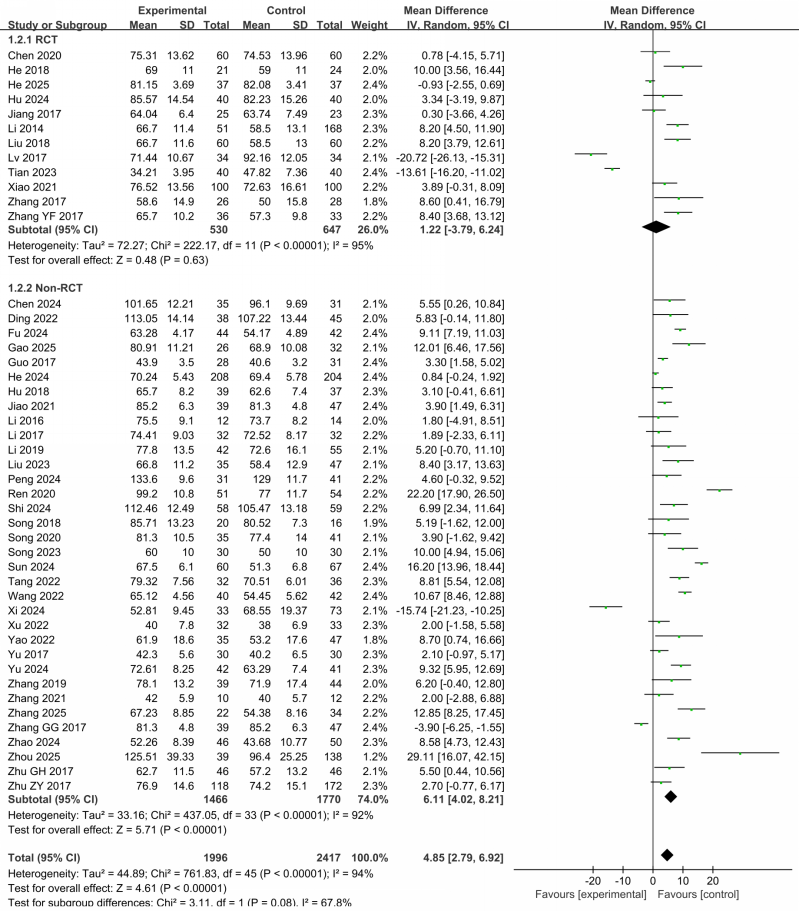


**Figure S4.** Subgroup analysis of operative time by study design.


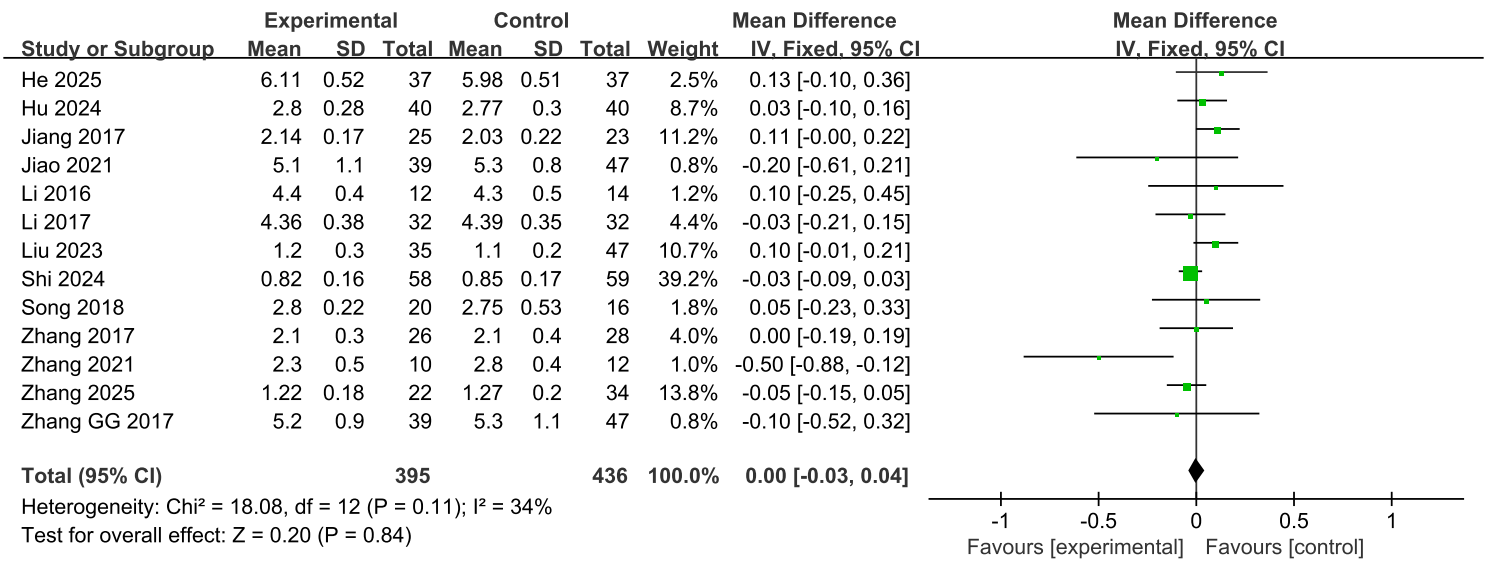


**Figure S5.** Forest plot for incision length.


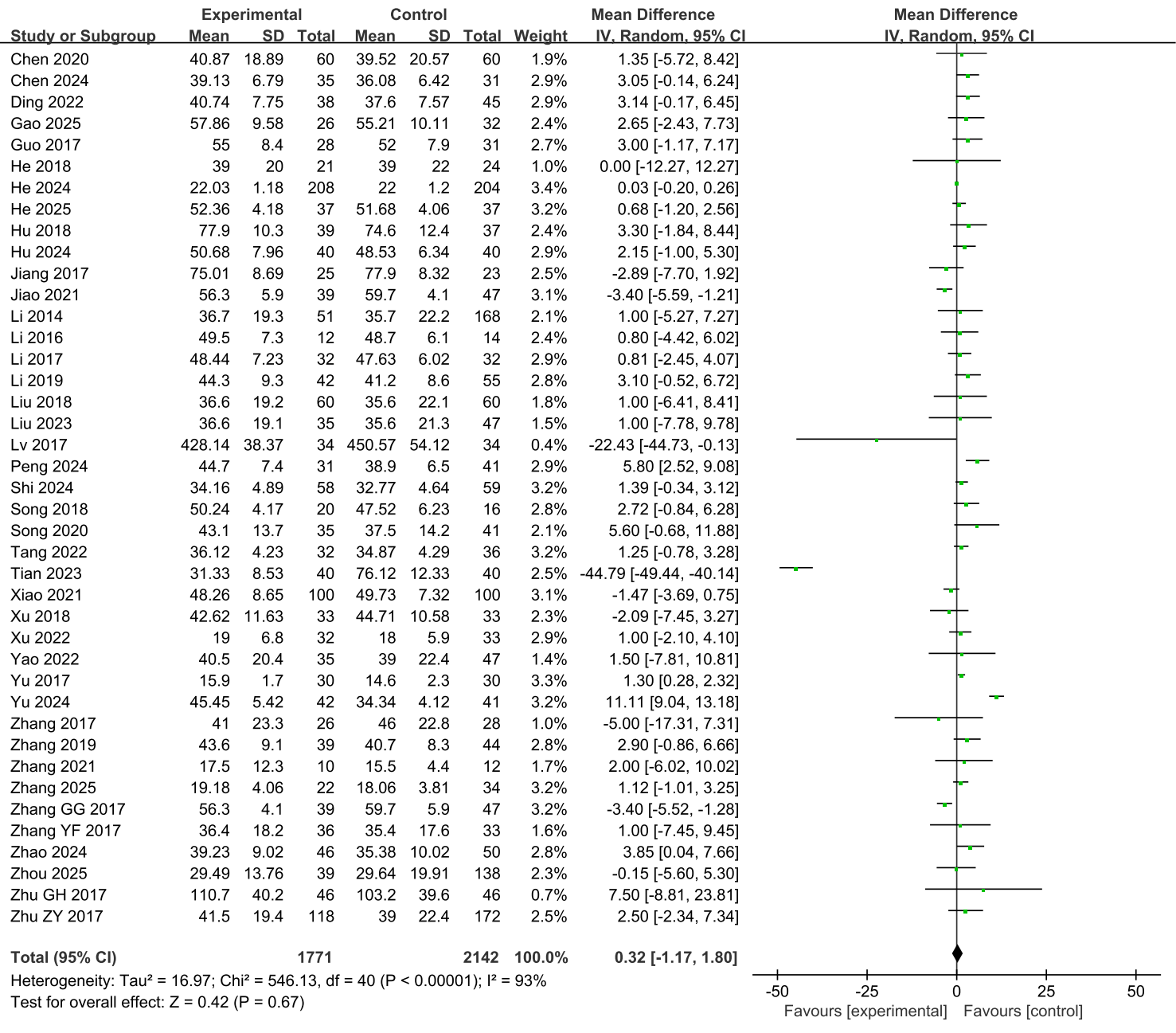


**Figure S6.** Forest plot for blood loss.


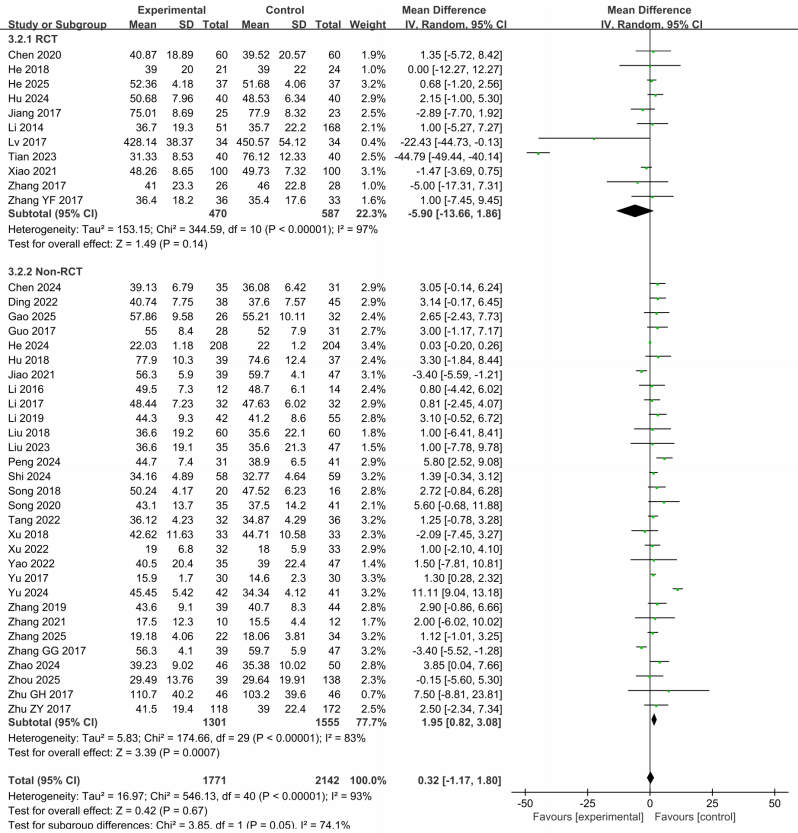


**Figure S7.** Subgroup analysis of blood loss by study design.


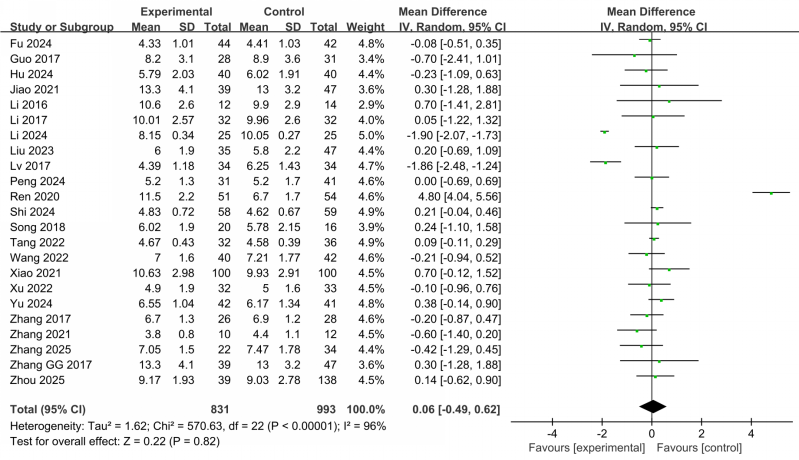


**Figure S8.** Forest plot for LOS.


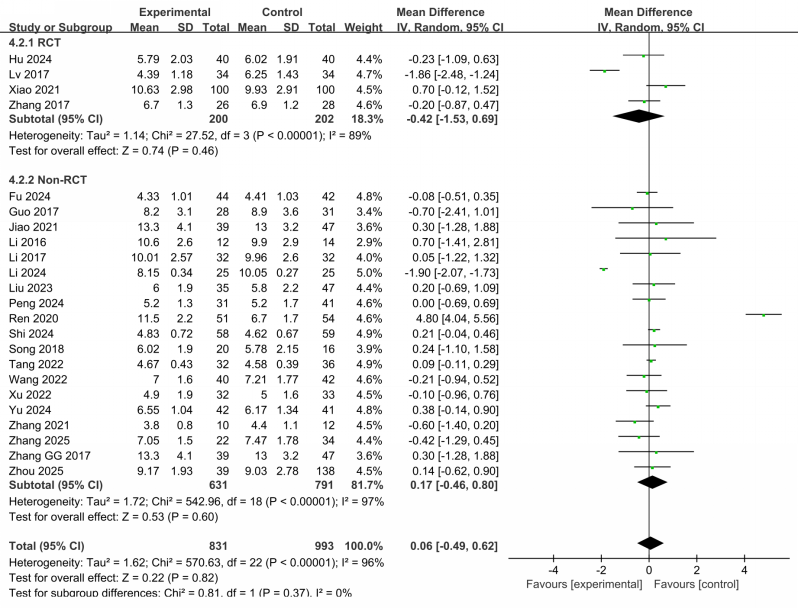


**Figure S9.** Subgroup analysis of LOS by study design.


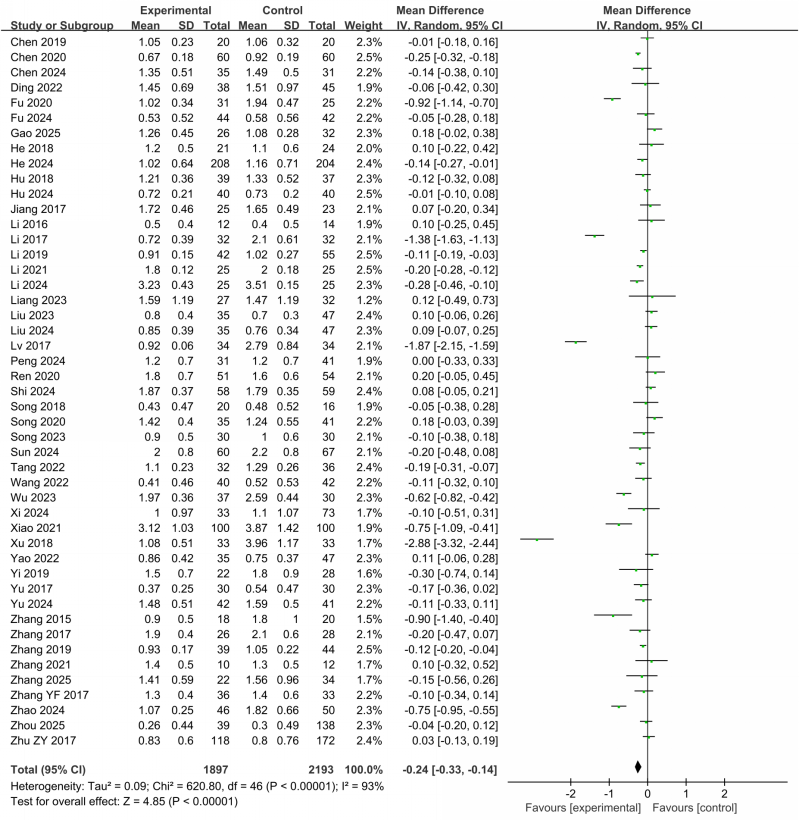

**Figure S10.** Forest plot of VAS score.


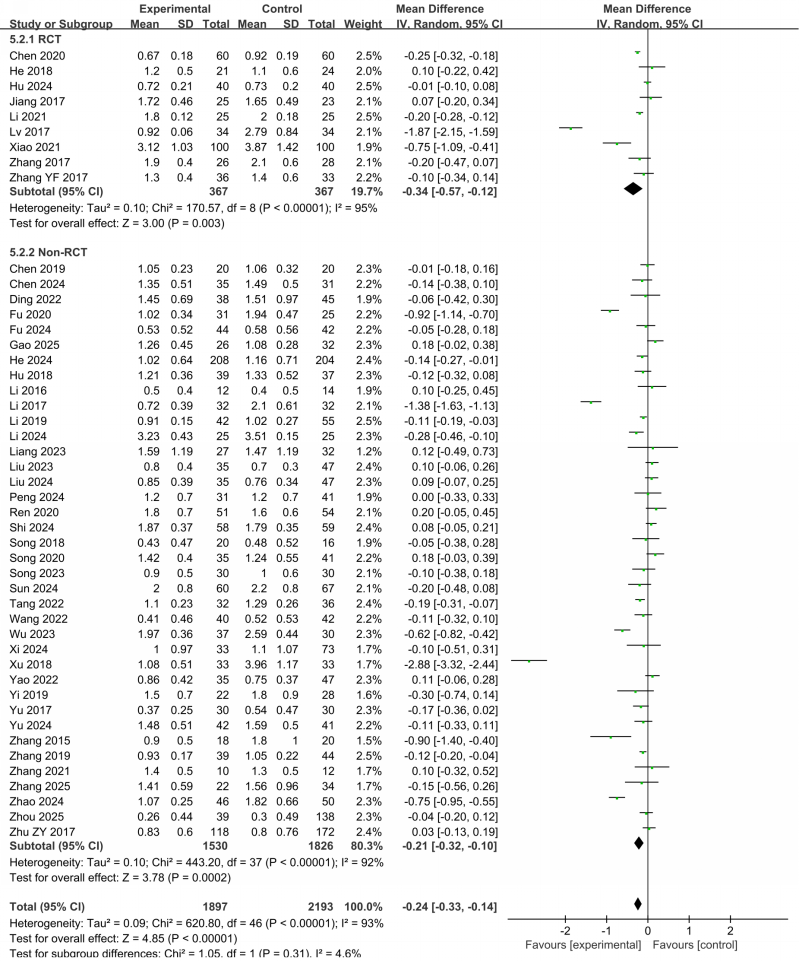


**Figure S11.** Subgroup analysis of VAS score by study design.


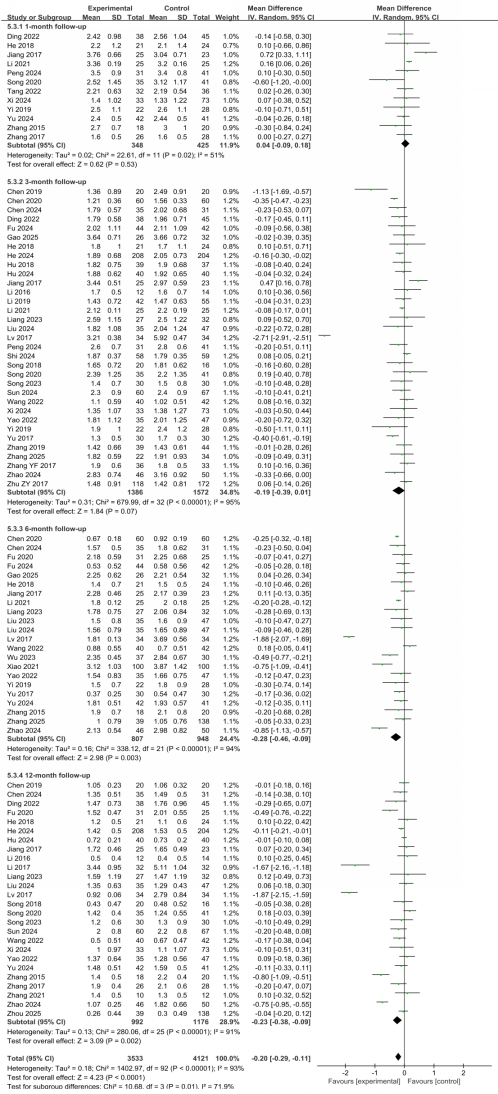


**Figure S12.** Subgroup analysis of VAS score by follow-up duration.


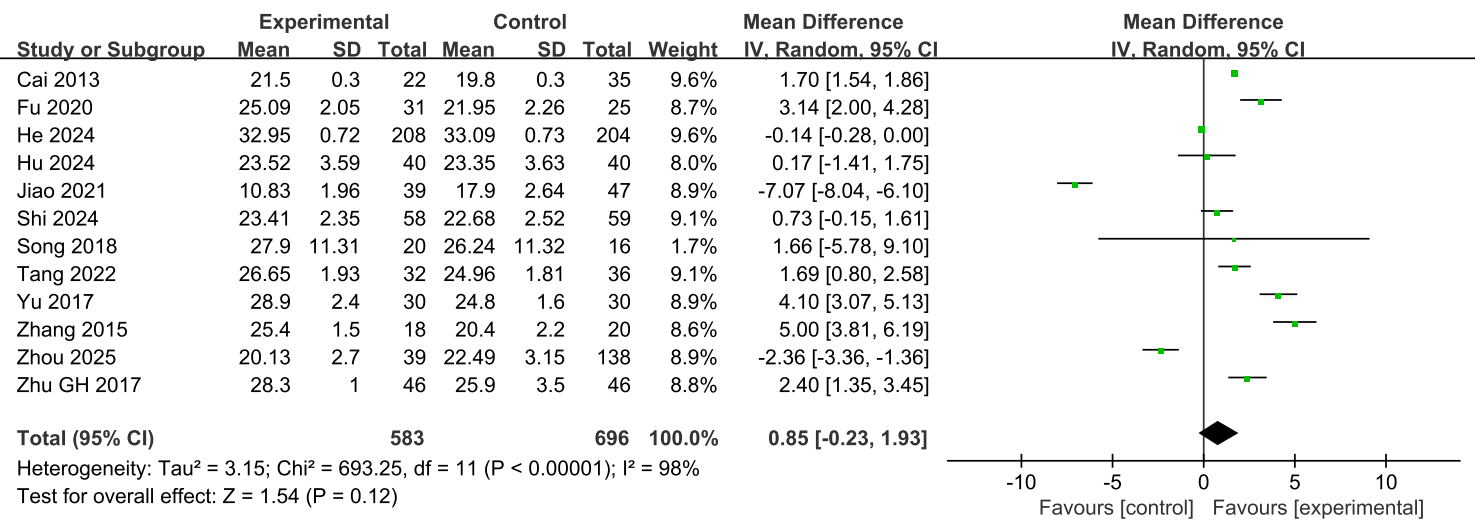


**Figure S13.** Forest plot of JOA score.


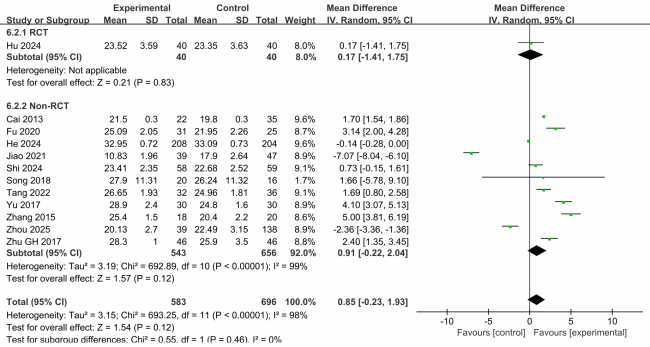


**Figure S14.** Subgroup analysis of JOA score by study design.


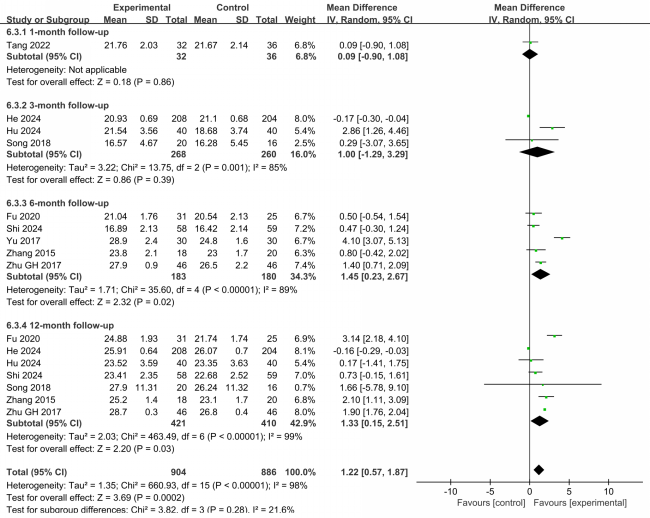


**Figure S15.** Subgroup analysis of JOA score by follow-up duration.


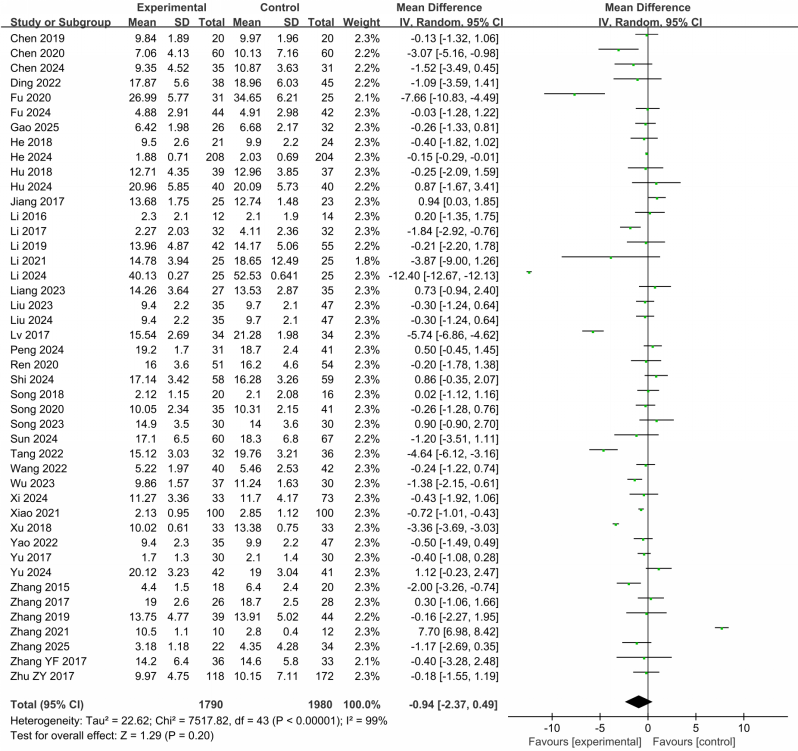


**Figure S16.** Forest plot of ODI score.


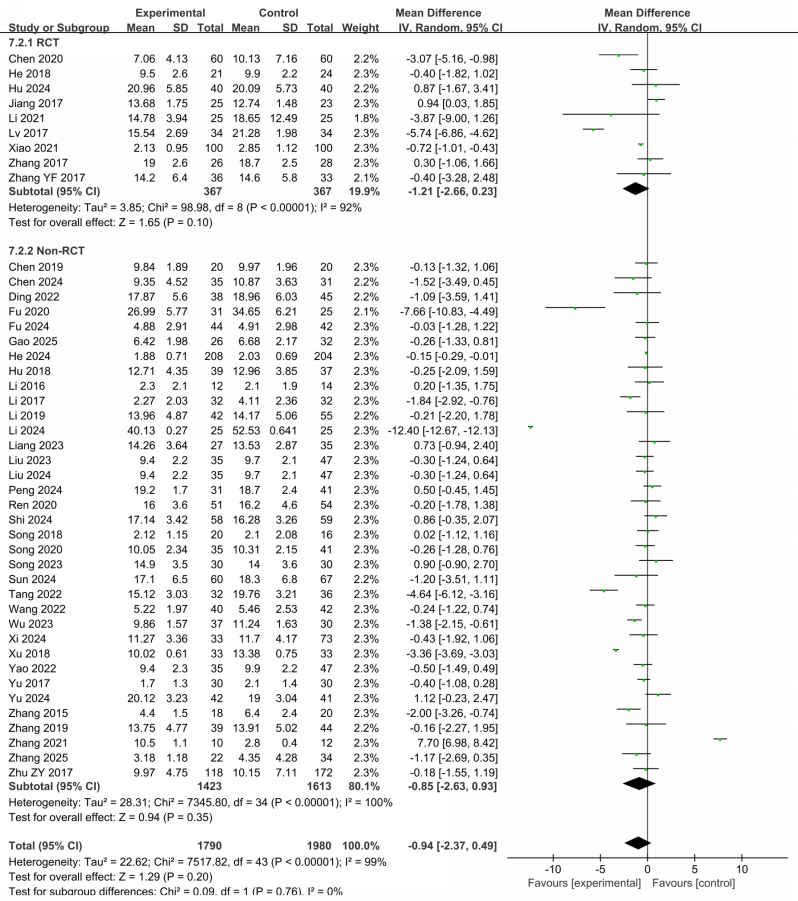


**Figure S17.** Subgroup analysis of ODI score by study design.


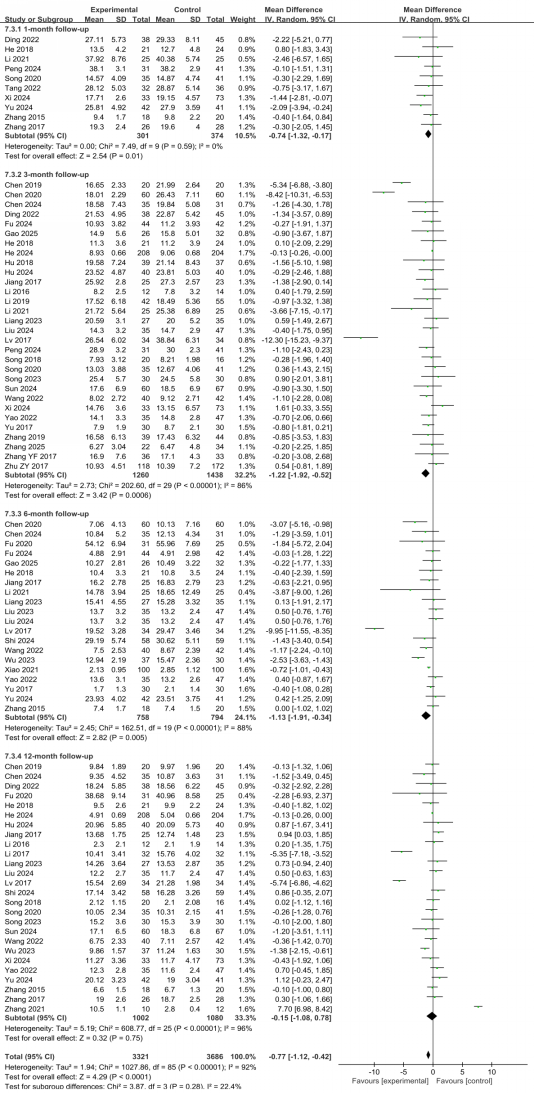


**Figure S18.** Subgroup analysis of ODI score by follow-up duration.


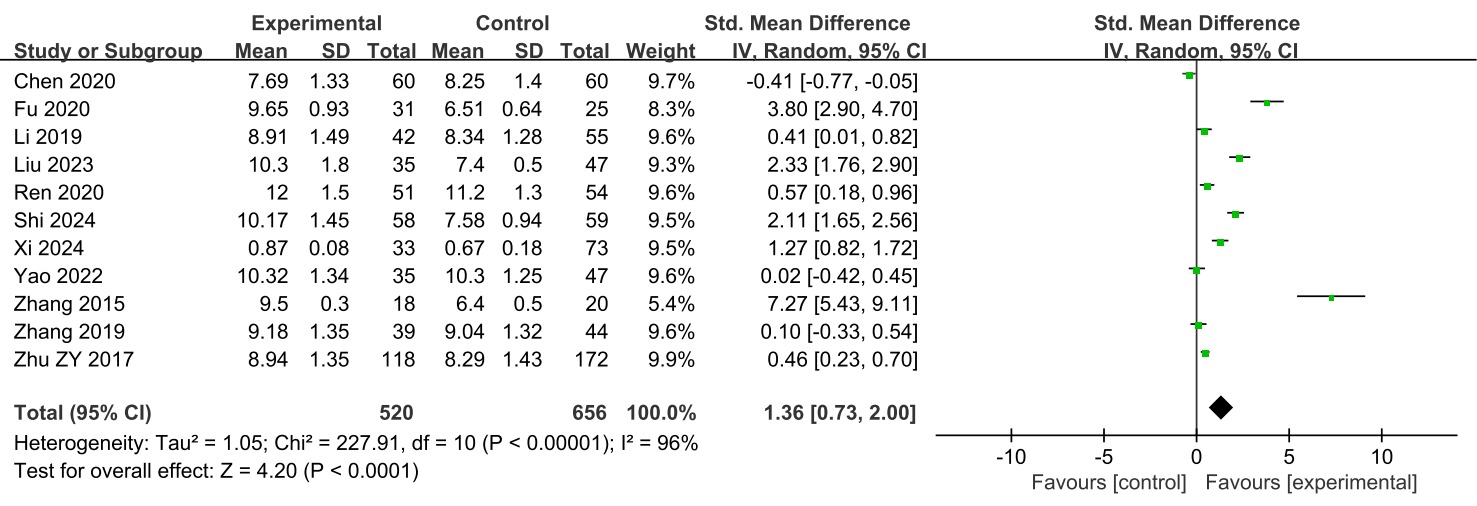


**Figure S19.** Forest plot for disc height.


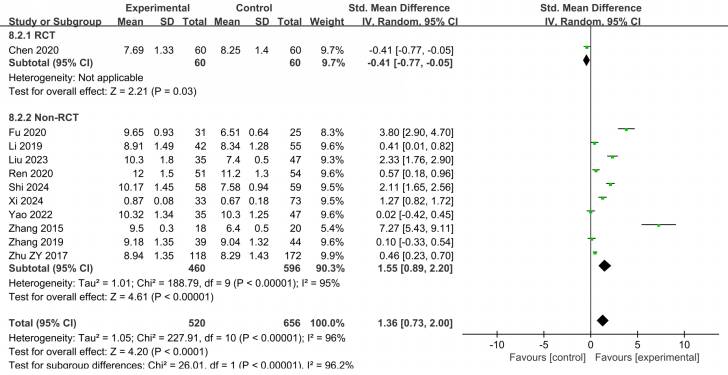


**Figure S20.** Subgroup analysis of disc height by study design.


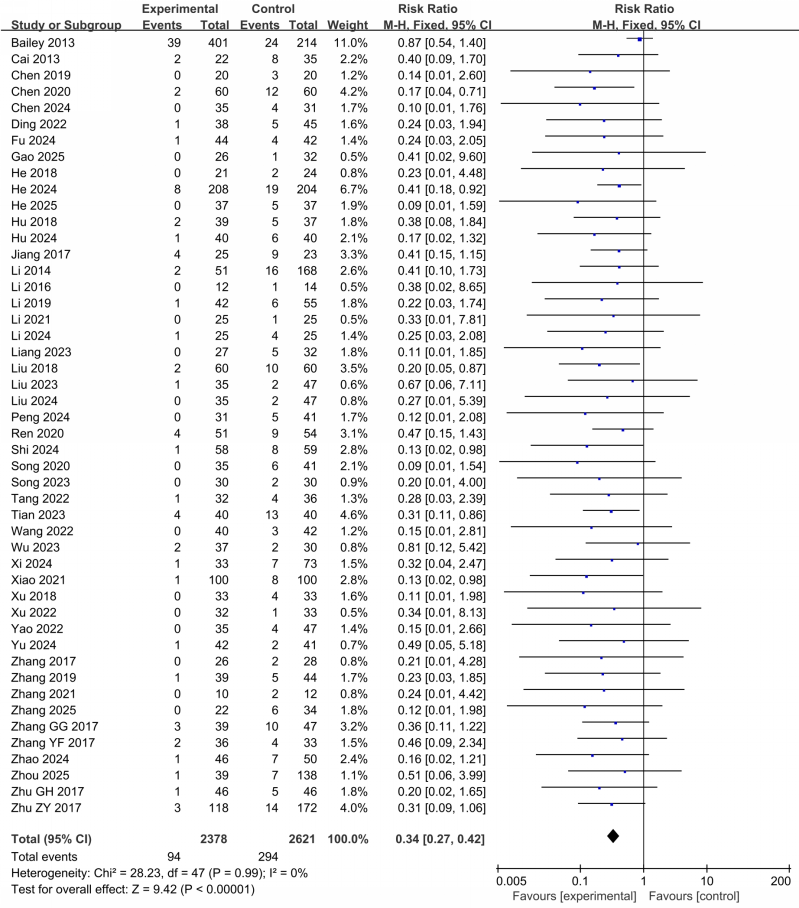


**Figure S21.** Forest plot for recurrence.


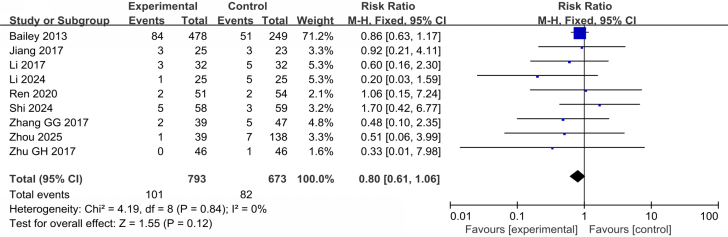


**Figure S22.** Forest plot for complication.

**Table S1**

Seneitivity analysis for operative time.

| Eliminated study | Heterogeneity | | Effect Model | MD | 95% CI | P Value |
| --- | --- | --- | --- | --- | --- | --- |
|  | P Value | I^2^ (%) |  |  |  |  |
| None | <0.00001 | 94 | Random | 4.85 | 2.79 to 6.92 | <0.00001 |
| Gao et al.,  2025 [45] | <0.00001 | 94 | Random | 4.70 | 2.62 to 6.78 | <0.00001 |
| He et al.,  2025 [46] | <0.00001 | 94 | Random | 5.00 | 2.87 to 7.14 | <0.00001 |
| Zhang et al.,  2025 [47] | <0.00001 | 94 | Random | 4.67 | 2.60 to 6.75 | <0.0001 |
| Zhou et al.,  2025 [48] | <0.00001 | 94 | Random | 4.55 | 2.49 to 6.61 | <0.0001 |
| Chen et al.,  2024 [49] | <0.00001 | 94 | Random | 4.84 | 2.75 to 6.93 | <0.00001 |
| Fu et al.,  2024 [50] | <0.00001 | 94 | Random | 4.75 | 2.64 to 6.86 | <0.00001 |
| He et al.,  2024 [51] | <0.00001 | 94 | Random | 4.98 | 2.75 to 7.20 | <0.0001 |
| Hu et al.,  2024 [52] | <0.00001 | 94 | Random | 4.89 | 2.80 to 6.98 | <0.00001 |
| Peng et al.,  2024 [55] | <0.00001 | 94 | Random | 4.86 | 2.77 to 6.96 | <0.00001 |
| Shi et al.,  2024 [56] | <0.00001 | 94 | Random | 4.81 | 2.71 to 6.90 | <0.00001 |
| Sun et al.,  2024 [57] | <0.00001 | 93 | Random | 4.55 | 2.59 to 6.51 | <0.00001 |
| Xi et al.,  2024 [58] | <0.00001 | 94 | Random | 5.29 | 3.26 to 7.32 | <0.00001 |
| Yu et al.,  2024 [59] | <0.00001 | 94 | Random | 4.75 | 2.66 to 6.84 | <0.00001 |
| Zhao et al.,  2024 [60] | <0.00001 | 94 | Random | 4.77 | 2.67 to 6.86 | <0.00001 |
| Liu et al.,  2023 [62] | <0.00001 | 94 | Random | 4.78 | 2.69 to 6.87 | <0.00001 |
| Song et al.,  2023 [63] | <0.00001 | 94 | Random | 4.74 | 2.65 to 6.83 | <0.00001 |
| Tian et al.,  2023 [64] | <0.00001 | 92 | Random | 5.28 | 3.40 to 7.15 | <0.00001 |
| Ding et al.,  2022 [66] | <0.00001 | 94 | Random | 4.84 | 2.74 to 6.93 | <0.00001 |
| Tang et al.,  2022 [67] | <0.00001 | 94 | Random | 4.76 | 2.67 to 6.86 | <0.00001 |
| Wang et al.,  2022 [68] | <0.00001 | 94 | Random | 4.71 | 2.63 to 6.79 | <0.00001 |
| Xu et al.,  2022 [69] | <0.00001 | 94 | Random | 4.92 | 2.82 to 7.03 | <0.00001 |
| Yao et al.,  2022 [70] | <0.00001 | 94 | Random | 4.78 | 2.70 to 6.87 | <0.00001 |
| Jiao et al.,  2021 [71] | <0.00001 | 94 | Random | 4.88 | 2.76 to 7.01 | <0.00001 |
| Xiao  2021 [73] | <0.00001 | 94 | Random | 4.88 | 2.78 to 6.98 | <0.00001 |
| Zhang et al.,  2021 [74] | <0.00001 | 94 | Random | 4.92 | 2.82 to 7.01 | <0.00001 |
| Chen  2020 [75] | <0.00001 | 94 | Random | 4.95 | 2.85 to 7.04 | <0.00001 |
| Ren et al.,  2020 [77] | <0.00001 | 94 | Random | 4.45 | 2.44 to 6.45 | <0.0001 |
| Song et al.,  2020 [78] | <0.00001 | 94 | Random | 4.88 | 2.78 to 6.97 | <0.00001 |
| Li  2019 [80] | <0.00001 | 94 | Random | 4.85 | 2.76 to 6.94 | <0.00001 |
| Zhang  2019 [82] | <0.00001 | 94 | Random | 4.83 | 2.74 to 6.92 | <0.00001 |
| He et al.,  2018 [83] | <0.00001 | 94 | Random | 4.75 | 2.67 to 6.84 | <0.00001 |
| Hu  2018 [84] | <0.00001 | 94 | Random | 4.90 | 2.79 to 7.01 | <0.00001 |
| Liu  2018 [85] | <0.00001 | 94 | Random | 4.78 | 2.69 to 6.87 | <0.00001 |
| Song et al.,  2018 [86] | <0.00001 | 94 | Random | 4.85 | 2.76 to 6.94 | <0.00001 |
| Guo et al.,  2017 [88] | <0.00001 | 94 | Random | 4.91 | 2.74 to 7.07 | <0.00001 |
| Jiang et al.,  2017 [89] | <0.00001 | 94 | Random | 4.96 | 2.86 to 7.06 | <0.00001 |
| Li et al.,  2017 [90] | <0.00001 | 94 | Random | 4.92 | 2.83 to 7.02 | <0.00001 |
| Lv et al.,  2017 [91] | <0.00001 | 94 | Random | 5.39 | 3.40 to 7.38 | <0.00001 |
| Yu et al.,  2017 [92] | <0.00001 | 94 | Random | 4.92 | 2.81 to 7.04 | <0.00001 |
| Zhang et al.,  2017 [93] | <0.00001 | 94 | Random | 4.79 | 2.70 to 6.87 | <0.00001 |
| Zhang et al.,  2017 [94] | <0.00001 | 94 | Random | 5.07 | 2.99 to 7.14 | <0.00001 |
| Zhang  2017 [95] | <0.00001 | 94 | Random | 4.78 | 2.68 to 6.87 | <0.00001 |
| Zhu et al.,  2017 [96] | <0.00001 | 94 | Random | 4.84 | 2.75 to 6.94 | <0.00001 |
| Zhu et al.,  2017 [97] | <0.00001 | 94 | Random | 4.91 | 2.80 to 7.02 | <0.00001 |
| Li et al.,  2016 [98] | <0.00001 | 94 | Random | 4.92 | 2.83 to 7.00 | <0.00001 |
| Li et al.,  2014 [100] | <0.00001 | 94 | Random | 4.78 | 2.68 to 6.87 | <0.00001 |
| Adolescent populations: He et al., 2025 [46]  Jiao et al., 2021 [71] Song et al., 2018 [86] Zhang et al., 2017 [94] Li et al., 2016 [98] | <0.00001 | 94 | Random | 5.35 | 3.06 to 7.63 | <0.00001 |
| High-risk studies:  Xu et al., 2022 [69]  Yao et al., 2022 [70]  Ren et al., 2020 [77]  Liu 2018 [85] | <0.00001 | 94 | Random | 4.34 | 2.24 to 6.44 | <0.0001 |

**Table S2**

Seneitivity analysis for blood loss.

| Eliminated study | Heterogeneity | | Effect Model | MD | 95% CI | P Value |
| --- | --- | --- | --- | --- | --- | --- |
|  | P Value | I^2^ (%) |  |  |  |  |
| None | <0.00001 | 93 | Random | 0.32 | -1.17 to 1.80 | 0.67 |
| Gao et al.,  2025 [45] | <0.00001 | 93 | Random | 0.26 | -1.25 to 1.76 | 0.74 |
| He et al.,  2025 [46] | <0.00001 | 93 | Random | 0.30 | -1.25 to 1.84 | 0.71 |
| Zhang et al.,  2025 [47] | <0.00001 | 93 | Random | 0.29 | -1.25 to 1.82 | 0.72 |
| Zhou et al.,  2025 [48] | <0.00001 | 93 | Random | 0.33 | -1.18 to 1.83 | 0.67 |
| Chen et al.,  2024 [49] | <0.00001 | 93 | Random | 0.23 | -1.28 to 1.75 | 0.76 |
| He et al.,  2024 [51] | <0.00001 | 93 | Random | 0.25 | -1.72 to 2.22 | 0.80 |
| Hu et al.,  2024 [52] | <0.00001 | 93 | Random | 0.26 | -1.26 to 1.78 | 0.74 |
| Peng et al.,  2024 [55] | <0.00001 | 93 | Random | 0.15 | -1.35 to 1.66 | 0.84 |
| Shi et al.,  2024 [56] | <0.00001 | 93 | Random | 0.27 | -1.28 to 1.82 | 0.73 |
| Yu et al.,  2024 [59] | <0.00001 | 91 | Random | -0.02 | -1.42 to 1.38 | 0.98 |
| Zhao et al.,  2024 [60] | <0.00001 | 93 | Random | 0.22 | -1.29 to 1.73 | 0.78 |
| Liu et al.,  2023 [62] | <0.00001 | 93 | Random | 0.31 | -1.19 to 1.80 | 0.69 |
| Tian et al.,  2023 [64] | <0.00001 | 79 | Random | 1.53 | 0.57 to 2.49 | 0.002 |
| Ding et al.,  2022 [66] | <0.00001 | 93 | Random | 0.23 | -1.28 to 1.75 | 0.76 |
| Tang et al.,  2022 [67] | <0.00001 | 93 | Random | 0.28 | -1.26 to 1.82 | 0.72 |
| Xu et al.,  2022 [69] | <0.00001 | 93 | Random | 0.29 | -1.23 to 1.81 | 0.70 |
| Yao et al.,  2022 [70] | <0.00001 | 93 | Random | 0.30 | -1.20 to 1.80 | 0.69 |
| Jiao et al.,  2021 [71] | <0.00001 | 93 | Random | 0.43 | -1.09 to 1.96 | 0.58 |
| Xiao  2021 [73] | <0.00001 | 93 | Random | 0.37 | -1.16 to 1.90 | 0.64 |
| Zhang et al.,  2021 [74] | <0.00001 | 93 | Random | 0.29 | -1.21 to 1.79 | 0.71 |
| Chen  2020 [75] | <0.00001 | 93 | Random | 0.30 | -1.20 to 1.80 | 0.70 |
| Song et al.,  2020 [78] | <0.00001 | 93 | Random | 0.20 | -1.30 to 1.70 | 0.79 |
| Li  2019 [80] | <0.00001 | 93 | Random | 0.24 | -1.28 to 1.75 | 0.76 |
| Zhang  2019 [82] | <0.00001 | 93 | Random | 0.24 | -1.27 to 1.75 | 0.75 |
| He et al.,  2018 [83] | <0.00001 | 93 | Random | 0.32 | -1.17 to 1.81 | 0.67 |
| Hu  2018 [84] | <0.00001 | 93 | Random | 0.24 | -1.26 to 1.75 | 0.75 |
| Liu  2018 [85] | <0.00001 | 93 | Random | 0.30 | -1.20 to 1.80 | 0.69 |
| Song et al.,  2018 [86] | <0.00001 | 93 | Random | 0.25 | -1.27 to 1.76 | 0.75 |
| Xu et al.,  2018 [87] | <0.00001 | 93 | Random | 0.37 | -1.13 to 1.88 | 0.63 |
| Guo et al.,  2017 [88] | <0.00001 | 93 | Random | 0.24 | -1.27 to 1.75 | 0.75 |
| Jiang et al.,  2017 [89] | <0.00001 | 93 | Random | 0.40 | -1.11 to 1.90 | 0.60 |
| Li et al.,  2017 [90] | <0.00001 | 93 | Random | 0.30 | -1.22 to 1.82 | 0.70 |
| Lv et al.,  2017 [91] | <0.00001 | 93 | Random | 0.41 | -1.08 to 1.89 | 0.59 |
| Yu et al.,  2017 [92] | <0.00001 | 93 | Random | 0.26 | -1.39 to 1.90 | 0.76 |
| Zhang et al.,  2017 [93] | <0.00001 | 93 | Random | 0.37 | -1.21 to 1.86 | 0.63 |
| Zhang et al.,  2017 [94] | <0.00001 | 93 | Random | 0.43 | -1.09 to 1.96 | 0.58 |
| Zhang  2017 [95] | <0.00001 | 93 | Random | 0.31 | -1.19 to 1.80 | 0.69 |
| Zhu et al.,  2017 [96] | <0.00001 | 93 | Random | 0.27 | -1.22 to 1.76 | 0.72 |
| Zhu et al.,  2017 [97] | <0.00001 | 93 | Random | 0.26 | -1.25 to 1.77 | 0.73 |
| Li et al.,  2016 [98] | <0.00001 | 93 | Random | 0.30 | -1.20 to 1.81 | 0.69 |
| Li et al.,  2014 [100] | <0.00001 | 93 | Random | 0.30 | -1.20 to 1.80 | 0.69 |
| Adolescent populations: He et al., 2025 [46]  Jiao et al., 2021 [71] Song et al., 2018 [86] Zhang et al., 2017 [94] Li et al., 2016 [98] | <0.00001 | 93 | Random | 0.46 | -1.25 to 2.17 | 0.60 |
| High-risk studies:  Xu et al., 2022 [69]  Yao et al., 2022 [70]  Liu 2018 [85] | <0.00001 | 93 | Random | 0.26 | -1.29 to 1.81 | 0.74 |

**Table S3**

Seneitivity analysis for LOS.

| Eliminated study | Heterogeneity | | Effect Model | MD | 95% CI | P Value |
| --- | --- | --- | --- | --- | --- | --- |
|  | P Value | I^2^ (%) |  |  |  |  |
| None | <0.00001 | 96 | Random | 0.06 | -0.49 to 0.62 | 0.82 |
| Zhang et al.,  2025 [47] | <0.00001 | 96 | Random | 0.09 | -0.49 to 0.66 | 0.77 |
| Zhou et al.,  2025 [48] | <0.00001 | 96 | Random | 0.06 | -0.51 to 0.63 | 0.84 |
| Fu et al.,  2024 [50] | <0.00001 | 96 | Random | 0.07 | -0.52 to 0.66 | 0.81 |
| Hu et al.,  2024 [52] | <0.00001 | 96 | Random | 0.08 | -0.50 to 0.65 | 0.79 |
| Li et al.,  2024 [53] | <0.00001 | 89 | Random | 0.16 | -0.25 to 0.58 | 0.44 |
| Peng et al.,  2024 [55] | <0.00001 | 96 | Random | 0.07 | -0.51 to 0.64 | 0.82 |
| Shi et al.,  2024 [56] | <0.00001 | 96 | Random | 0.06 | -0.55 to 0.66 | 0.85 |
| Yu et al.,  2024 [59] | <0.00001 | 96 | Random | 0.05 | -0.53 to 0.62 | 0.87 |
| Liu et al.,  2023 [62] | <0.00001 | 96 | Random | 0.06 | -0.52 to 0.63 | 0.85 |
| Tang et al.,  2022 [67] | <0.00001 | 96 | Random | 0.06 | -0.57 to 0.70 | 0.84 |
| Wang et al.,  2022 [68] | <0.00001 | 96 | Random | 0.08 | -0.50 to 0.65 | 0.79 |
| Xu et al.,  2022 [69] | <0.00001 | 96 | Random | 0.07 | -0.50 to 0.64 | 0.81 |
| Jiao et al.,  2021 [71] | <0.00001 | 96 | Random | 0.05 | -0.51 to 0.62 | 0.85 |
| Xiao  2021 [73] | <0.00001 | 96 | Random | 0.03 | -0.54 to 0.60 | 0.91 |
| Zhang et al.,  2021 [74] | <0.00001 | 96 | Random | 0.09 | -0.48 to 0.67 | 0.75 |
| Ren et al.,  2020 [77] | <0.00001 | 94 | Random | -0.17 | -0.65 to 0.30 | 0.48 |
| Song et al.,  2018 [86] | <0.00001 | 96 | Random | 0.06 | -0.51 to 0.62 | 0.85 |
| Guo et al.,  2017 [88] | <0.00001 | 96 | Random | 0.09 | -0.48 to 0.66 | 0.76 |
| Li et al.,  2017 [90] | <0.00001 | 96 | Random | 0.06 | -0.51 to 0.63 | 0.83 |
| Lv et al.,  2017 [91] | <0.00001 | 96 | Random | 0.16 | -0.41 to 0.73 | 0.59 |
| Zhang et al.,  2017 [93] | <0.00001 | 96 | Random | 0.08 | -0.50 to 0.65 | 0.80 |
| Zhang et al.,  2017 [94] | <0.00001 | 96 | Random | 0.05 | -0.51 to 0.62 | 0.85 |
| Li et al.,  2016 [98] | <0.00001 | 96 | Random | 0.04 | -0.52 to 0.61 | 0.88 |
| Adolescent populations:  Jiao et al., 2021 [71] Song et al., 2018 [86] Zhang et al., 2017 [94] Li et al., 2016 [98] | <0.00001 | 97 | Random | 0.01 | -0.59 to 0.62 | 0.96 |
| High-risk studies:  Xu et al., 2022 [69]  Ren et al., 2020 [77] | <0.00001 | 95 | Random | -0.18 | -0.67 to 0.32 | 0.48 |

**Table S4**

Seneitivity analysis for VAS score.

| Eliminated study | Heterogeneity | | Effect Model | MD | 95% CI | P Value |
| --- | --- | --- | --- | --- | --- | --- |
|  | P Value | I^2^ (%) |  |  |  |  |
| None | <0.00001 | 93 | Random | -0.24 | -0.33 to -0.14 | <0.00001 |
| Gao et al.,  2025 [45] | <0.00001 | 93 | Random | -0.25 | -0.34 to -0.15 | <0.00001 |
| Zhang et al.,  2025 [47] | <0.00001 | 93 | Random | -0.24 | -0.33 to -0.14 | <0.00001 |
| Zhou et al.,  2025 [48] | <0.00001 | 93 | Random | -0.24 | -0.34 to -0.14 | <0.00001 |
| Chen et al.,  2024 [49] | <0.00001 | 93 | Random | -0.24 | -0.34 to -0.14 | <0.00001 |
| Fu et al.,  2024 [50] | <0.00001 | 93 | Random | -0.24 | -0.34 to -0.14 | <0.00001 |
| He et al.,  2024 [51] | <0.00001 | 93 | Random | -0.24 | -0.34 to -0.14 | <0.00001 |
| Hu et al.,  2024 [52] | <0.00001 | 93 | Random | -0.24 | -0.34 to -0.14 | <0.00001 |
| Li et al.,  2024 [53] | <0.00001 | 93 | Random | -0.24 | -0.33 to -0.14 | <0.00001 |
| Liu et al.,  2024 [54] | <0.00001 | 93 | Random | -0.24 | -0.34 to -0.15 | <0.00001 |
| Peng et al.,  2024 [55] | <0.00001 | 93 | Random | -0.24 | -0.34 to -0.14 | <0.00001 |
| Shi et al.,  2024 [56] | <0.00001 | 93 | Random | -0.24 | -0.34 to -0.15 | <0.00001 |
| Sun et al.,  2024 [57] | <0.00001 | 93 | Random | -0.24 | -0.33 to -0.14 | <0.00001 |
| Xi et al.,  2024 [58] | <0.00001 | 93 | Random | -0.24 | -0.34 to -0.14 | <0.00001 |
| Yu et al.,  2024 [59] | <0.00001 | 93 | Random | -0.24 | -0.34 to -0.15 | <0.00001 |
| Zhao et al.,  2024 [60] | <0.00001 | 92 | Random | -0.22 | -0.32 to -0.13 | <0.00001 |
| Liang et al.,  2023 [61] | <0.00001 | 93 | Random | -0.24 | -0.34 to -0.14 | <0.00001 |
| Liu et al.,  2023 [62] | <0.00001 | 93 | Random | -0.24 | -0.34 to -0.15 | <0.00001 |
| Song et al.,  2023 [63] | <0.00001 | 93 | Random | -0.24 | -0.34 to -0.14 | <0.00001 |
| Wu et al.,  2023 [65] | <0.00001 | 92 | Random | -0.23 | -0.32 to -0.13 | <0.00001 |
| Ding et al.,  2022 [66] | <0.00001 | 93 | Random | -0.24 | -0.34 to -0.14 | <0.00001 |
| Tang et al.,  2022 [67] | <0.00001 | 93 | Random | -0.24 | -0.34 to -0.14 | <0.00001 |
| Wang et al.,  2022 [68] | <0.00001 | 93 | Random | -0.24 | -0.34 to -0.14 | <0.00001 |
| Yao et al.,  2022 [70] | <0.00001 | 93 | Random | -0.24 | -0.34 to -0.15 | <0.00001 |
| Li et al.,  2021 [72] | <0.00001 | 93 | Random | -0.24 | -0.34 to -0.14 | <0.00001 |
| Xiao  2021 [73] | <0.00001 | 93 | Random | -0.23 | -0.32 to -0.13 | <0.00001 |
| Zhang et al.,  2021 [74] | <0.00001 | 93 | Random | -0.24 | -0.34 to -0.15 | <0.00001 |
| Chen  2020 [75] | <0.00001 | 93 | Random | -0.24 | -0.34 to -0.14 | <0.00001 |
| Fu et al.,  2020 [76] | <0.00001 | 92 | Random | -0.22 | -0.31 to -0.13 | <0.00001 |
| Ren et al.,  2020 [77] | <0.00001 | 93 | Random | -0.25 | -0.34 to -0.15 | <0.00001 |
| Song et al.,  2020 [78] | <0.00001 | 93 | Random | -0.25 | -0.34 to -0.15 | <0.00001 |
| Chen et al.,  2019 [79] | <0.00001 | 93 | Random | -0.24 | -0.34 to -0.14 | <0.00001 |
| Li  2019 [80] | <0.00001 | 93 | Random | -0.24 | -0.34 to -0.14 | <0.00001 |
| Yi et al.,  2019 [81] | <0.00001 | 93 | Random | -0.24 | -0.33 to -0.14 | <0.00001 |
| Zhang  2019 [82] | <0.00001 | 93 | Random | -0.24 | -0.34 to -0.14 | <0.00001 |
| He et al.,  2018 [83] | <0.00001 | 93 | Random | -0.24 | -0.34 to -0.15 | <0.00001 |
| Hu  2018 [84] | <0.00001 | 93 | Random | -0.24 | -0.34 to -0.14 | <0.00001 |
| Song et al.,  2018 [86] | <0.00001 | 93 | Random | -0.24 | -0.34 to -0.14 | <0.00001 |
| Xu et al.,  2018 [87] | <0.00001 | 90 | Random | -0.19 | -0.28 to -0.11 | <0.0001 |
| Jiang et al.,  2017 [89] | <0.00001 | 93 | Random | -0.24 | -0.34 to -0.15 | <0.00001 |
| Li et al.,  2017 [90] | <0.00001 | 92 | Random | -0.21 | -0.30 to -0.12 | <0.00001 |
| Lv et al.,  2017 [91] | <0.00001 | 91 | Random | -0.20 | -0.28 to -0.11 | <0.00001 |
| Yu et al.,  2017 [92] | <0.00001 | 93 | Random | -0.24 | -0.34 to -0.14 | <0.00001 |
| Zhang et al.,  2017 [93] | <0.00001 | 93 | Random | -0.24 | -0.33 to -0.14 | <0.00001 |
| Zhang  2017 [95] | <0.00001 | 93 | Random | -0.24 | -0.34 to -0.14 | <0.00001 |
| Zhu et al.,  2017 [97] | <0.00001 | 93 | Random | -0.24 | -0.34 to -0.15 | <0.00001 |
| Li et al.,  2016 [98] | <0.00001 | 93 | Random | -0.24 | -0.34 to -0.15 | <0.00001 |
| Zhang et al.,  2015 [99] | <0.00001 | 93 | Random | -0.23 | -0.32 to -0.13 | <0.00001 |
| Adolescent populations: Wu et al., 2023 [65]  Song et al., 2018 [86]  Li et al., 2016 [98] | <0.00001 | 93 | Random | -0.24 | -0.34 to -0.14 | <0.00001 |
| High-risk studies:  Yao et al., 2022 [70]  Ren et al., 2020 [77] | <0.00001 | 93 | Random | -0.25 | -0.35 to -0.16 | <0.00001 |

**Table S5**

Seneitivity analysis for JOA score.

| Eliminated study | Heterogeneity | | Effect Model | MD | 95% CI | P Value |
| --- | --- | --- | --- | --- | --- | --- |
|  | P Value | I^2^ (%) |  |  |  |  |
| None | <0.00001 | 98 | Random | 0.85 | -0.23 to 1.93 | 0.12 |
| Zhou et al.,  2025 [48] | <0.00001 | 98 | Random | 1.16 | 0.04 to 2.28 | 0.04 |
| He et al.,  2024 [51] | <0.00001 | 98 | Random | 0.97 | -0.71 to 2.66 | 0.26 |
| Hu et al.,  2024 [52] | <0.00001 | 99 | Random | 0.91 | -0.22 to 2.04 | 0.12 |
| Shi et al.,  2024 [56] | <0.00001 | 99 | Random | 0.86 | -0.29 to 2.01 | 0.14 |
| Tang et al.,  2022 [67] | <0.00001 | 99 | Random | 0.77 | -0.38 to 1.91 | 0.19 |
| Jiao et al.,  2021 [71] | <0.00001 | 98 | Random | 1.62 | 0.68 to 2.56 | 0.0007 |
| Fu et al.,  2020 [76] | <0.00001 | 99 | Random | 0.63 | -0.50 to 1.76 | 0.27 |
| Song et al.,  2018 [86] | <0.00001 | 99 | Random | 0.84 | -0.26 to 1.93 | 0.13 |
| Yu et al.,  2017 [92] | <0.00001 | 98 | Random | 0.53 | -0.58 to 1.64 | 0.35 |
| Zhu et al.,  2017 [96] | <0.00001 | 99 | Random | 0.70 | -0.44 to 1.84 | 0.23 |
| Zhang et al.,  2015 [99] | <0.00001 | 98 | Random | 0.46 | -0.64 to 1.56 | 0.42 |
| Cai et al.,  2013 [102] | <0.00001 | 98 | Random | 0.78 | -0.86 to 2.43 | 0.35 |
| Adolescent populations:  Jiao et al., 2021 [71]  Song et al., 2018 [86] | <0.00001 | 98 | Random | 1.62 | 0.67 to 2.57 | 0.0008 |

**Table S6**

Seneitivity analysis for ODI score.

| Eliminated study | Heterogeneity | | Effect Model | MD | 95% CI | P Value |
| --- | --- | --- | --- | --- | --- | --- |
|  | P Value | I^2^ (%) |  |  |  |  |
| None | <0.00001 | 99 | Random | -0.94 | -2.37 to 0.49 | 0.20 |
| Gao et al.,  2025 [45] | <0.00001 | 99 | Random | -0.96 | -2.41 to 0.50 | 0.20 |
| Zhang et al.,  2025 [47] | <0.00001 | 99 | Random | -0.93 | -2.38 to 0.51 | 0.21 |
| Chen et al.,  2024 [49] | <0.00001 | 99 | Random | -0.93 | -2.37 to 0.52 | 0.21 |
| Fu et al.,  2024 [50] | <0.00001 | 99 | Random | -0.96 | -2.41 to 0.49 | 0.19 |
| He et al.,  2024 [51] | <0.00001 | 99 | Random | -0.96 | -2.64 to 0.71 | 0.26 |
| Hu et al.,  2024 [52] | <0.00001 | 99 | Random | -0.98 | -2.42 to 0.46 | 0.18 |
| Li et al.,  2024 [53] | <0.00001 | 96 | Random | -0.58 | -1.19 to 0.03 | 0.06 |
| Liu et al.,  2024 [54] | <0.00001 | 99 | Random | -0.95 | -2.41 to 0.50 | 0.20 |
| Peng et al.,  2024 [55] | <0.00001 | 99 | Random | -0.97 | -2.43 to 0.48 | 0.19 |
| Shi et al.,  2024 [56] | <0.00001 | 99 | Random | -0.98 | -2.43 to 0.47 | 0.18 |
| Sun et al.,  2024 [57] | <0.00001 | 99 | Random | -0.93 | -2.38 to 0.51 | 0.21 |
| Xi et al.,  2024 [58] | <0.00001 | 99 | Random | -0.95 | -2.40 to 0.50 | 0.20 |
| Yu et al.,  2024 [59] | <0.00001 | 99 | Random | -0.99 | -2.44 to 0.46 | 0.18 |
| Liang et al.,  2023 [61] | <0.00001 | 99 | Random | -0.98 | -2.42 to 0.47 | 0.19 |
| Liu et al.,  2023 [62] | <0.00001 | 99 | Random | -0.95 | -2.41 to 0.50 | 0.20 |
| Song et al.,  2023 [63] | <0.00001 | 99 | Random | -0.98 | -2.43 to 0.46 | 0.18 |
| Wu et al.,  2023 [65] | <0.00001 | 99 | Random | -0.93 | -2.39 to 0.53 | 0.21 |
| Ding et al.,  2022 [66] | <0.00001 | 99 | Random | -0.94 | -2.38 to 0.51 | 0.20 |
| Tang et al.,  2022 [67] | <0.00001 | 99 | Random | -0.85 | -2.30 to 0.59 | 0.25 |
| Wang et al.,  2022 [68] | <0.00001 | 99 | Random | -0.96 | -2.41 to 0.50 | 0.20 |
| Yao et al.,  2022 [70] | <0.00001 | 99 | Random | -0.95 | -2.40 to 0.50 | 0.20 |
| Li et al.,  2021 [72] | <0.00001 | 99 | Random | -0.89 | -2.33 to 0.56 | 0.23 |
| Xiao  2021 [73] | <0.00001 | 99 | Random | -0.95 | -2.52 to 0.62 | 0.24 |
| Zhang et al.,  2021 [74] | <0.00001 | 99 | Random | -1.14 | -2.54 to 0.25 | 0.11 |
| Chen  2020 [75] | <0.00001 | 99 | Random | -0.89 | -2.34 to 0.56 | 0.23 |
| Fu et al.,  2020 [76] | <0.00001 | 99 | Random | -0.79 | -2.24 to 0.65 | 0.28 |
| Ren et al.,  2020 [77] | <0.00001 | 99 | Random | -0.96 | -2.40 to 0.49 | 0.20 |
| Song et al.,  2020 [78] | <0.00001 | 99 | Random | -0.96 | -2.41 to 0.50 | 0.20 |
| Chen et al.,  2019 [79] | <0.00001 | 99 | Random | -0.96 | -2.41 to 0.49 | 0.20 |
| Li  2019 [80] | <0.00001 | 99 | Random | -0.96 | -2.40 to 0.49 | 0.20 |
| Zhang  2019 [82] | <0.00001 | 99 | Random | -0.96 | -2.40 to 0.49 | 0.19 |
| He et al.,  2018 [83] | <0.00001 | 99 | Random | -0.95 | -2.40 to 0.50 | 0.20 |
| Hu  2018 [84] | <0.00001 | 99 | Random | -0.96 | -2.40 to 0.49 | 0.20 |
| Song et al.,  2018 [86] | <0.00001 | 99 | Random | -0.96 | -2.41 to 0.49 | 0.19 |
| Xu et al.,  2018 [87] | <0.00001 | 99 | Random | -0.88 | -2.42 to 0.65 | 0.26 |
| Jiang et al.,  2017 [89] | <0.00001 | 99 | Random | -0.98 | -2.44 to 0.47 | 0.18 |
| Li et al.,  2017 [90] | <0.00001 | 99 | Random | -0.92 | -2.37 to 0.53 | 0.22 |
| Lv et al.,  2017 [91] | <0.00001 | 99 | Random | -0.83 | -2.27 to 0.62 | 0.26 |
| Yu et al.,  2017 [92] | <0.00001 | 99 | Random | -0.95 | -2.42 to 0.51 | 0.20 |
| Zhang et al.,  2017 [93] | <0.00001 | 99 | Random | -0.97 | -2.42 to 0.48 | 0.19 |
| Zhang  2017 [95] | <0.00001 | 99 | Random | -0.95 | -2.39 to 0.49 | 0.20 |
| Zhu et al.,  2017 [97] | <0.00001 | 99 | Random | -0.96 | -2.41 to 0.49 | 0.20 |
| Li et al.,  2016 [98] | <0.00001 | 99 | Random | -0.97 | -2.41 to 0.48 | 0.19 |
| Zhang et al.,  2015 [99] | <0.00001 | 99 | Random | -0.91 | -2.36 to 0.54 | 0.22 |
| Adolescent populations:  Wu et al., 2023 [65]  Song et al., 2018 [86]  Li et al., 2016 [98] | <0.00001 | 99 | Random | -0.98 | -2.49 to 0.53 | 0.20 |
| High-risk studies:  Yao et al., 2022 [70]  Ren et al., 2020 [77] | <0.00001 | 99 | Random | -0.97 | -2.44 to 0.51 | 0.20 |

**Table S7**

Seneitivity analysis for disc height.

| Eliminated study | Heterogeneity | | Effect Model | SMD | 95% CI | P Value |
| --- | --- | --- | --- | --- | --- | --- |
|  | P Value | I^2^ (%) |  |  |  |  |
| None | <0.00001 | 96 | Random | 1.36 | 0.73 to 2.00 | <0.0001 |
| Shi et al.,  2024 [56] | <0.00001 | 95 | Random | 1.26 | 0.63 to 1.90 | 0.0001 |
| Xi et al.,  2024 [58] | <0.00001 | 96 | Random | 1.38 | 0.69 to 2.08 | <0.0001 |
| Liu et al.,  2023 [62] | <0.00001 | 95 | Random | 1.24 | 0.61 to 1.88 | <0.0001 |
| Yao et al.,  2022 [70] | <0.00001 | 96 | Random | 1.52 | 0.83 to 2.21 | <0.0001 |
| Chen  2020 [75] | <0.00001 | 95 | Random | 1.55 | 0.89 to 2.20 | <0.00001 |
| Fu et al.,  2020 [76] | <0.00001 | 95 | Random | 1.11 | 0.50 to 1.71 | 0.0003 |
| Ren et al.,  2020 [77] | <0.00001 | 96 | Random | 1.47 | 0.76 to 2.18 | <0.0001 |
| Li  2019 [80] | <0.00001 | 96 | Random | 1.48 | 0.73 to 2.19 | <0.0001 |
| Zhang  2019 [82] | <0.00001 | 96 | Random | 1.51 | 0.82 to 2.20 | <0.0001 |
| Zhu et al.,  2017 [97] | <0.00001 | 96 | Random | 1.51 | 0.74 to 2.28 | 0.0001 |
| Zhang et al.,  2015 [99] | <0.00001 | 95 | Random | 1.01 | 0.43 to 1.59 | 0.0007 |
| High-risk studies:  Yao et al., 2022 [70]  Ren et al., 2020 [77] | <0.00001 | 96 | Random | 1.66 | 0.87 to 2.44 | <0.0001 |

**
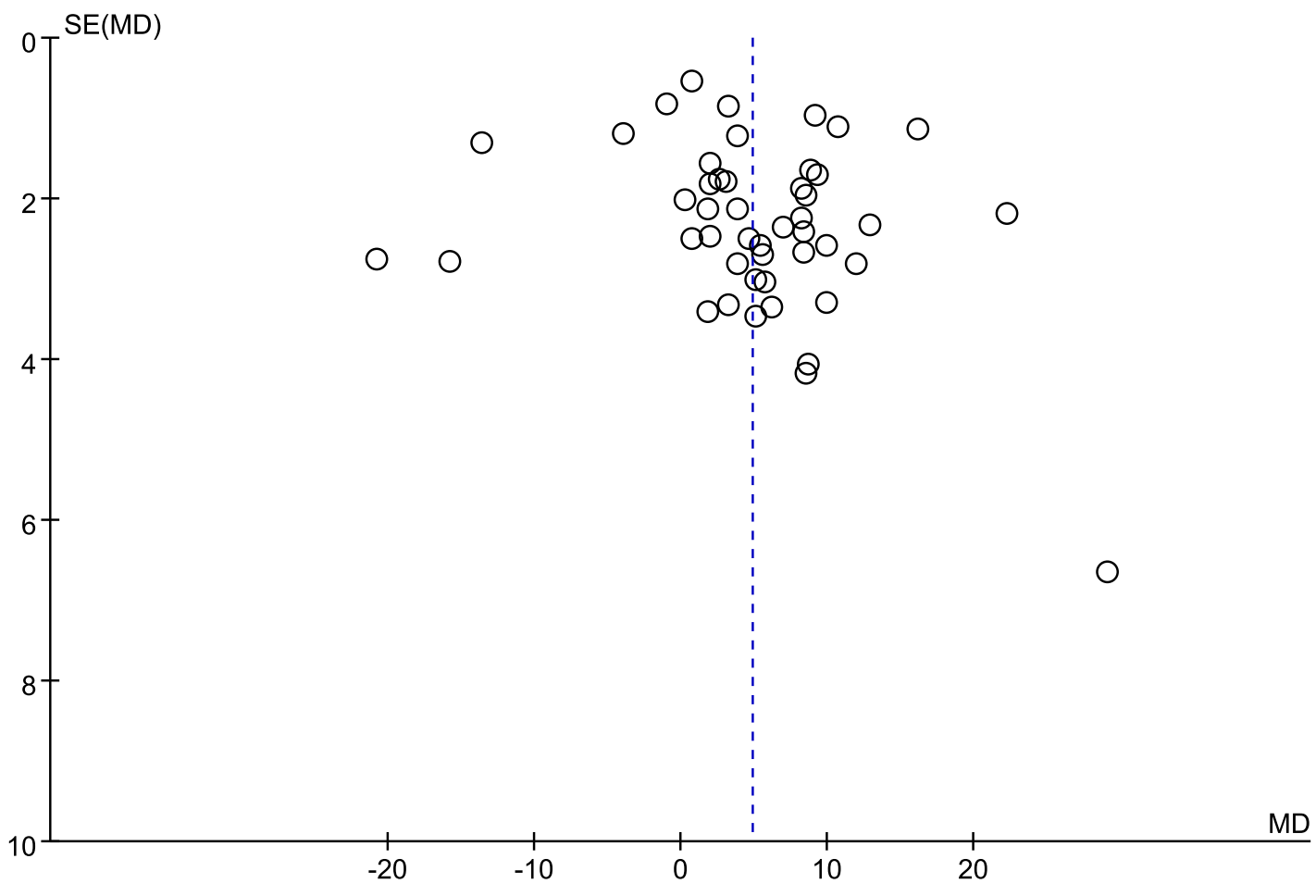
**

**Figure S23.** The funnel plot of operative time.


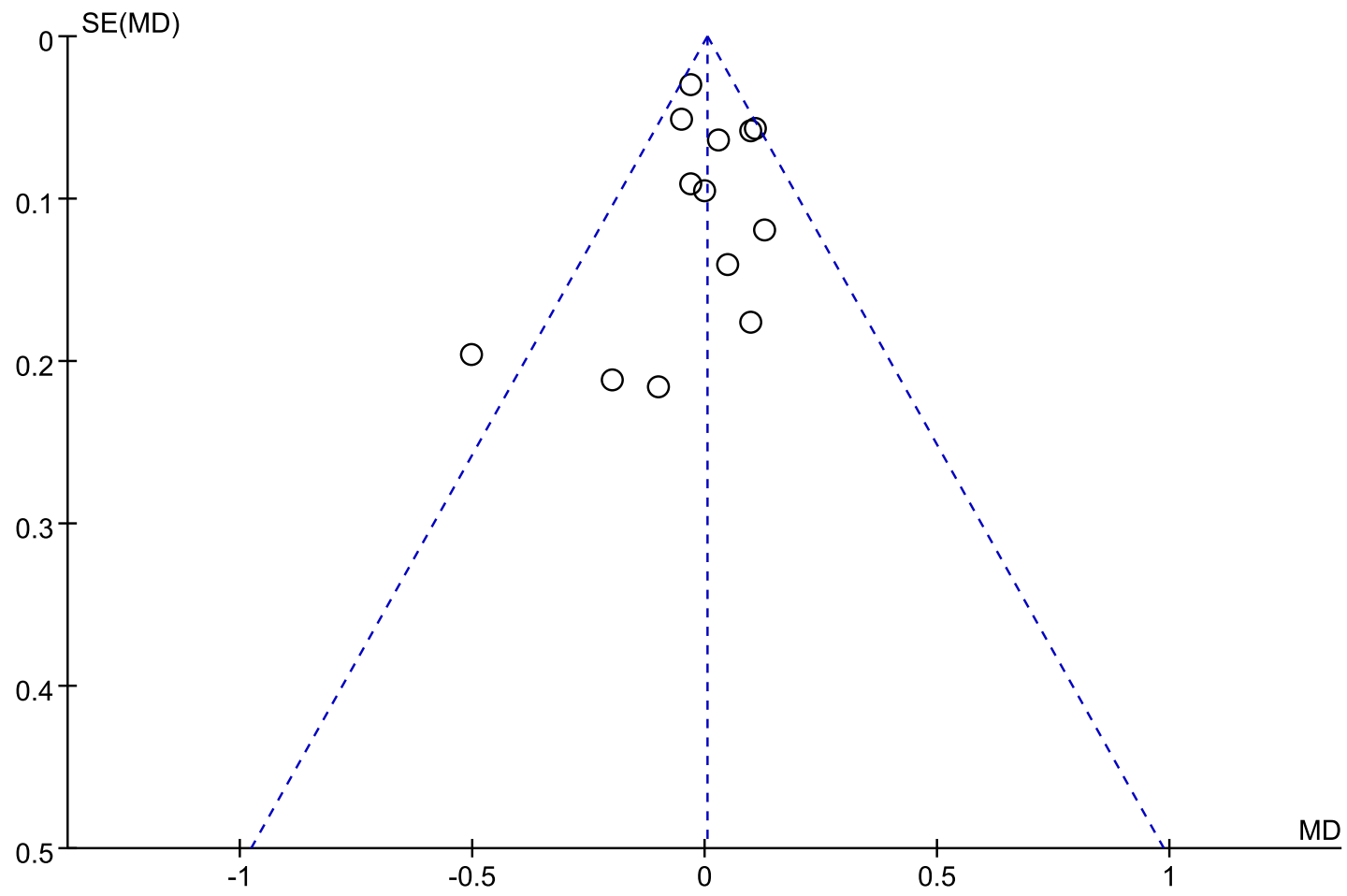


**Figure S24.** The funnel plot of incision length.


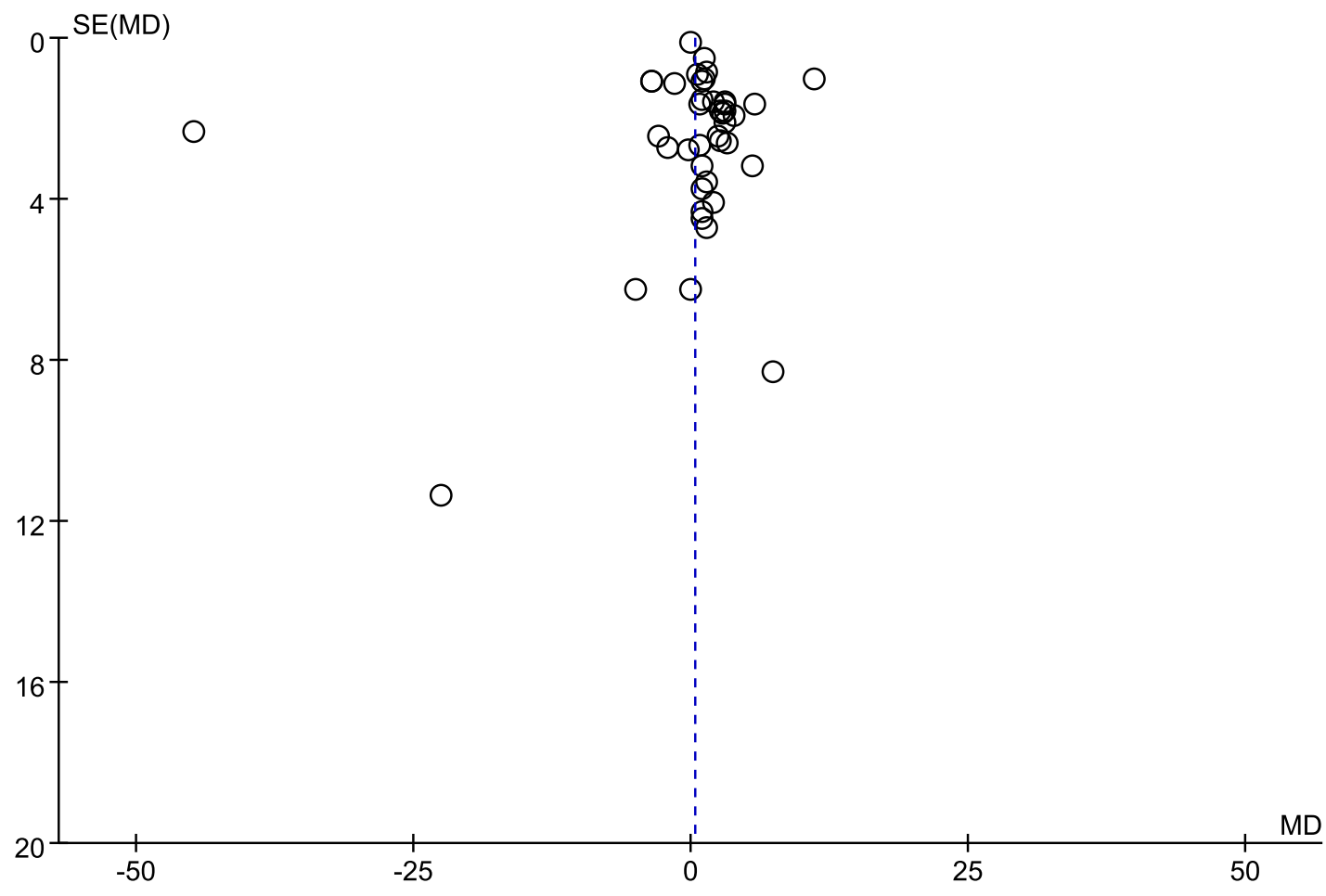


**Figure S25.** The funnel plot of blood loss.


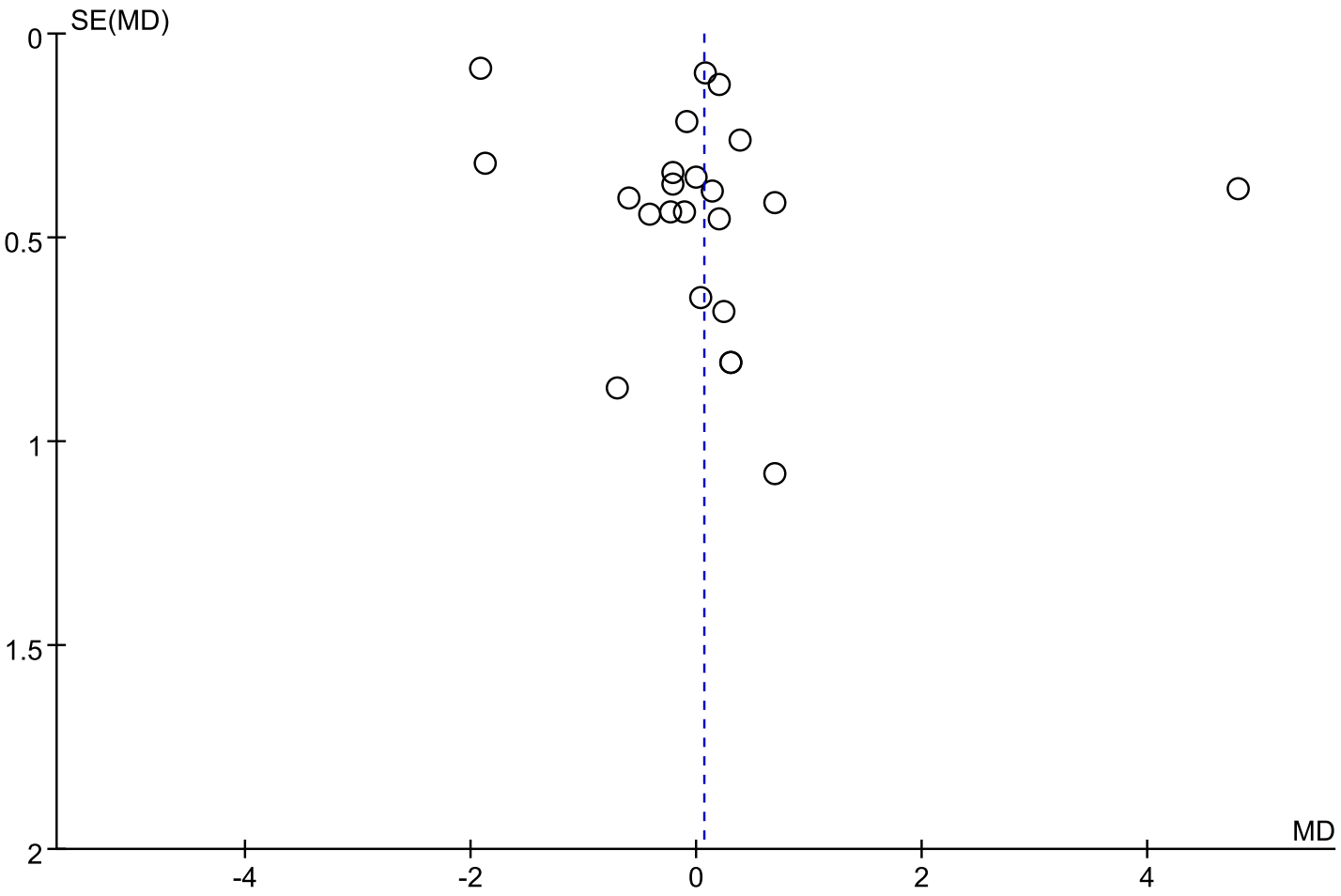


**Figure S26.** The funnel plot of LOS.


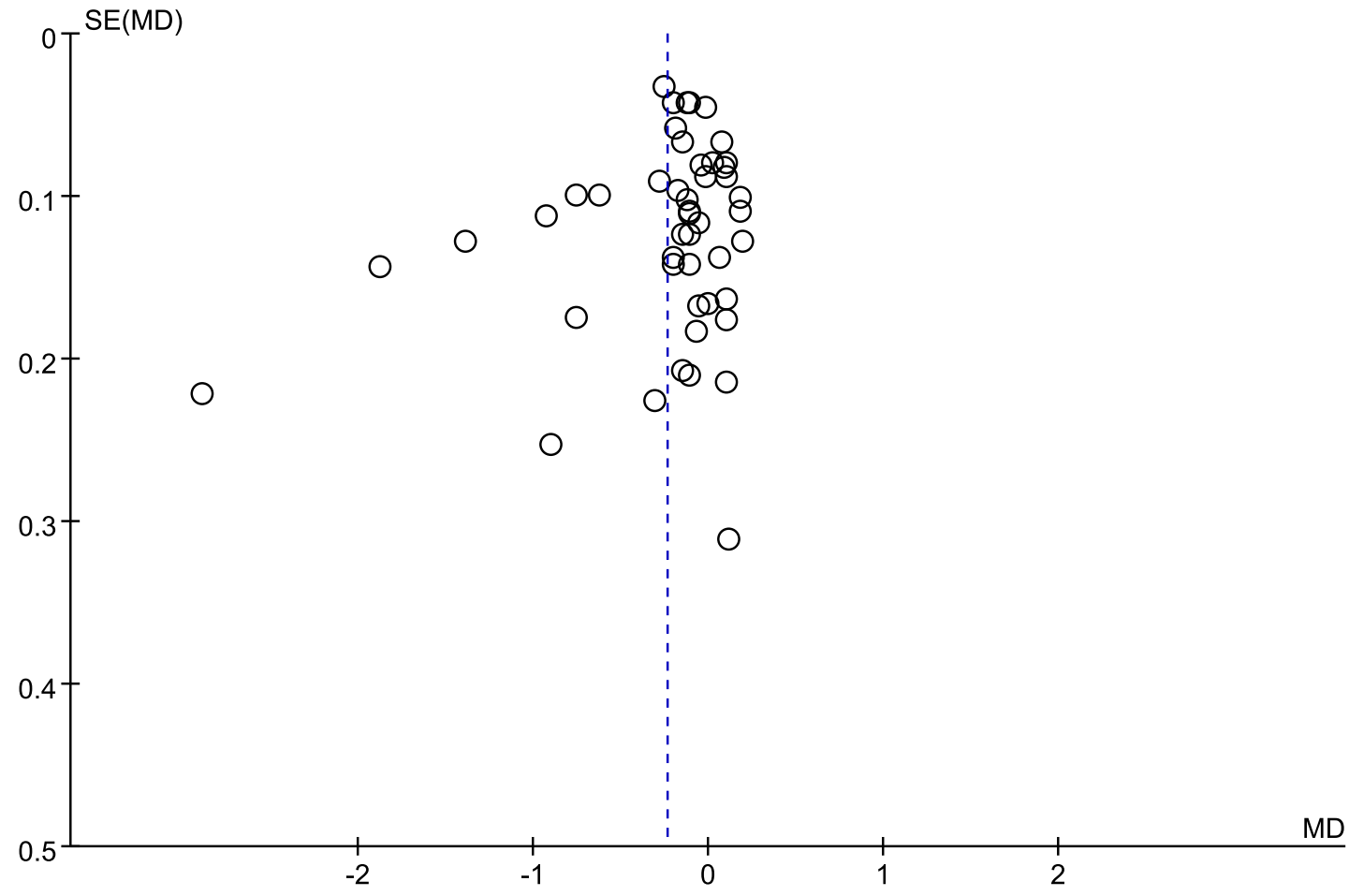


**Figure S27.** The funnel plot of VAS score.


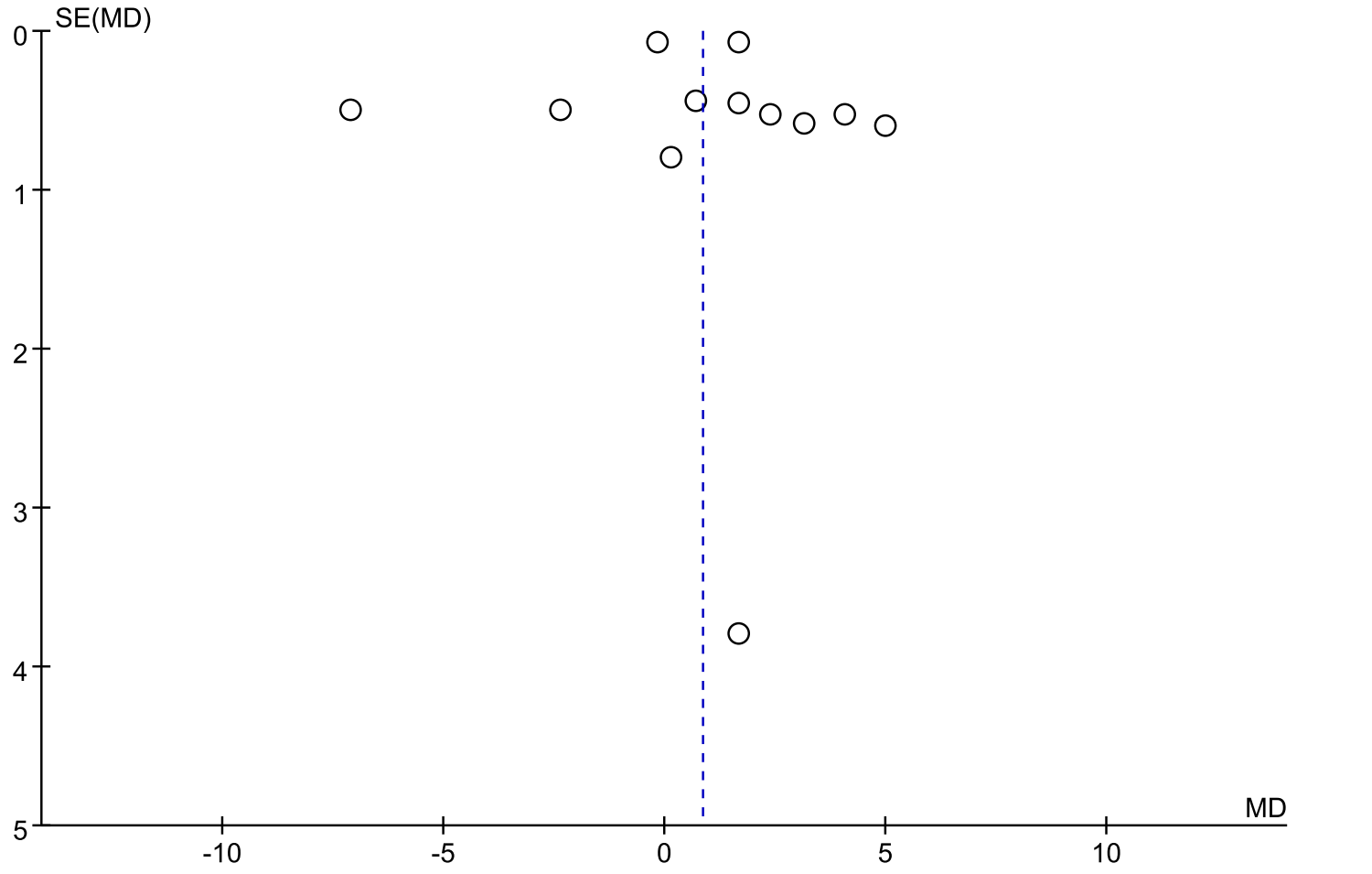


**Figure S28.** The funnel plot of JOA score.


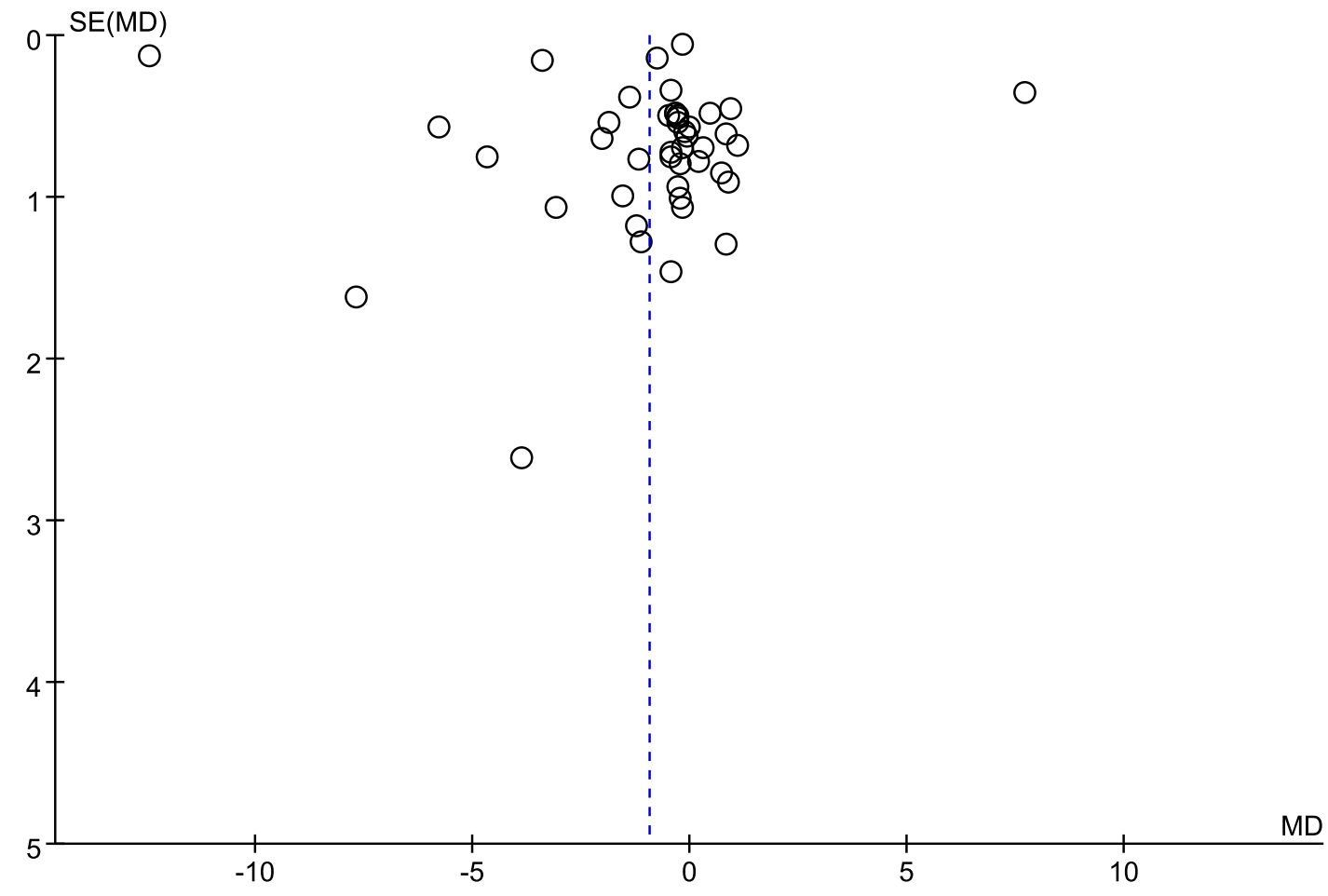


**Figure S29.** The funnel plot of ODI score.


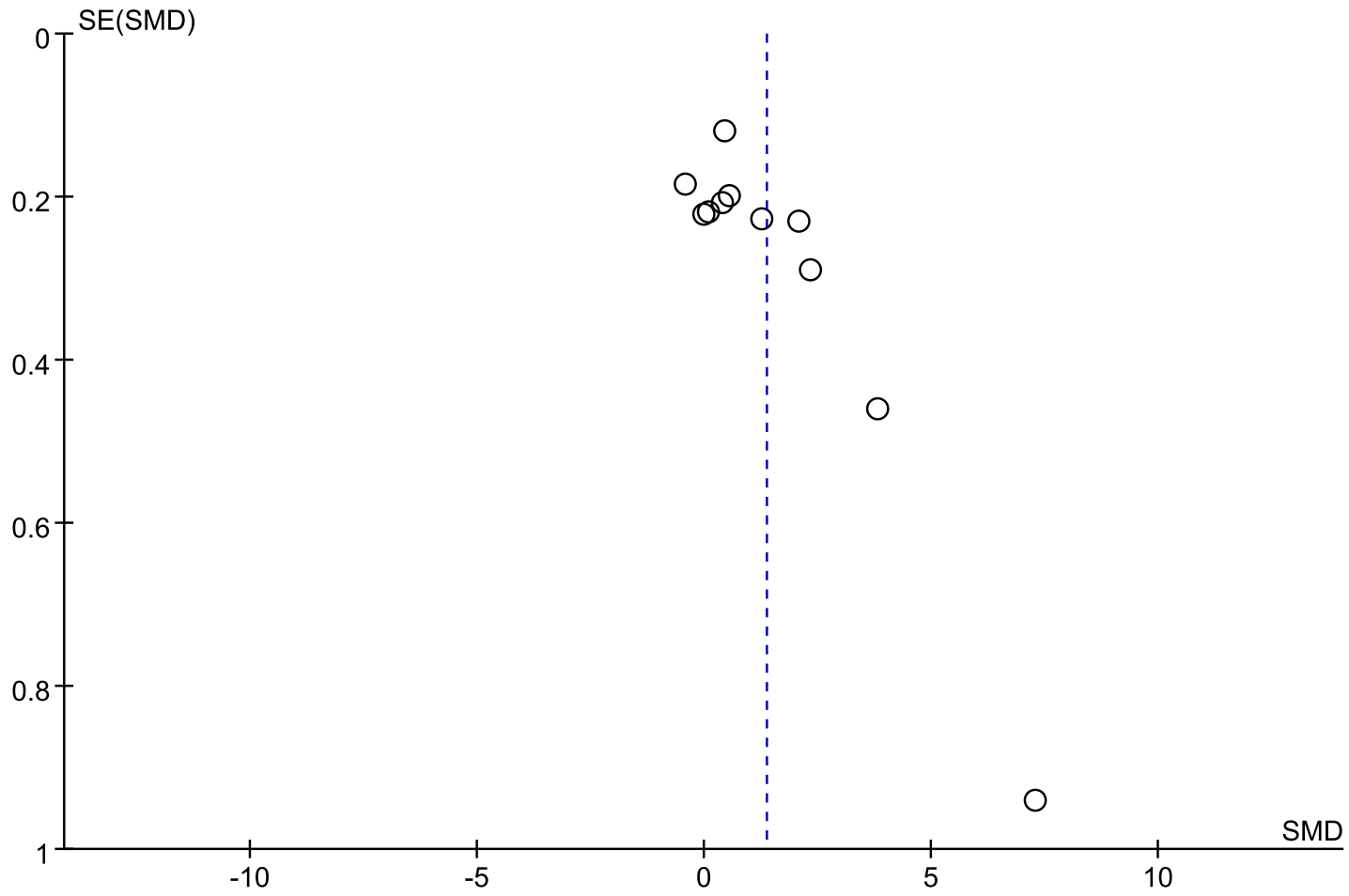


**Figure S30.** The funnel plot of disc height.


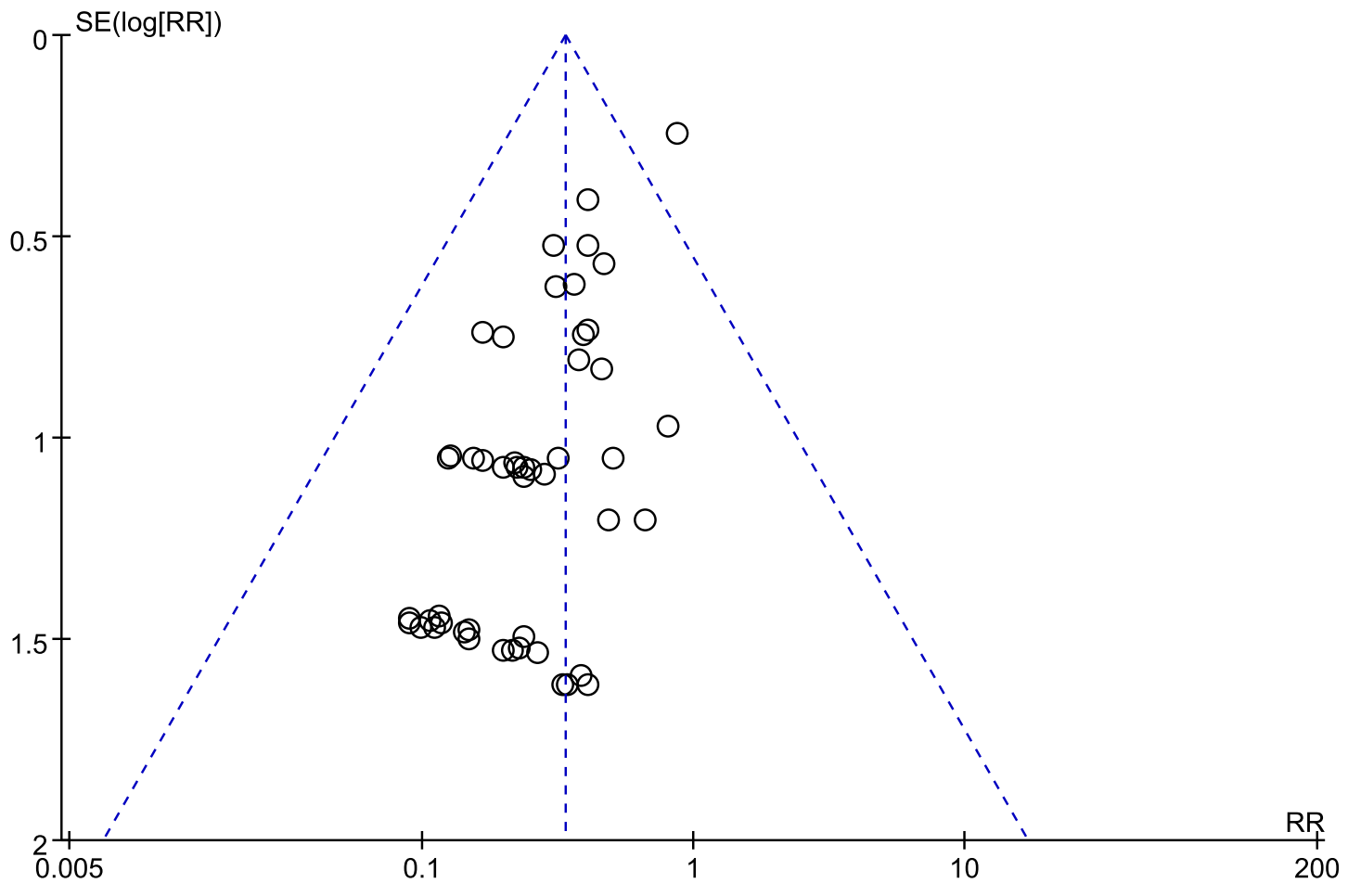


**Figure S31.** The funnel plot of recurrence.

**Table S8**

Publication bias of the included studies.

| Outcomes | Number of trails | Egger’s test (P value) |
| --- | --- | --- |
| Operative time | 46 studies | 0.199 |
| Incision length | 13 studies | 0.766 |
| Blood loss | 41 studies | 0.751 |
| LOS | 23 studies | 0.083 |
| VAS score | 47 studies | 0.186 |
| JOA score | 12 studies | 0.878 |
| ODI score | 44 studies | 0.545 |
| Disc height | 11 studies | 0.013 |
| Recurrence | 48 studies | 0.000 |

**Table S9**

GRADE evaluation of evidence quality.

| Outcome, number of sties  (number of participants) | Study design | I^2^ (%) | Study design ^1^ | Risk of bias ^2^ | Inconsistency of results ^3^ | Imprecision ^4^ | Imprecision ^5^ | Publication bias ^6^ | Overall quality of evidence |
| --- | --- | --- | --- | --- | --- | --- | --- | --- | --- |
| Operative time  46 studies (4413) | RCT and Non-RCT | 94 | -1 | -1 | -1 | 0 | 0 | 0 | Very low |
| Incision length  13 studies (831) | RCT and Non-RCT | 34 | -1 | 0 | 0 | 0 | 0 | 0 | Moderate |
| blood loss  41 studies (3913) | RCT and Non-RCT | 93 | -1 | -1 | -1 | 0 | 0 | 0 | Very low |
| LOS  23 studies (1824) | RCT and Non-RCT | 96 | -1 | -1 | -1 | 0 | 0 | 0 | Very low |
| VAS score  47 studies (4090) | RCT and Non-RCT | 93 | -1 | -1 | -1 | 0 | 0 | 0 | Very low |
| JOA score  12 studies (1279) | RCT and Non-RCT | 98 | -1 | 0 | -1 | 0 | 0 | 0 | Low |
| ODI score  44 studies (3770) | RCT and Non-RCT | 99 | -1 | -1 | -1 | 0 | 0 | 0 | Very low |
| Disc height  11 studies (1176) | RCT and Non-RCT | 96 | -1 | -1 | -1 | 0 | 0 | -1 | Very low |
| Recurrence  48 studies (4999) | RCT and Non-RCT | 0 | -1 | -1 | 0 | 0 | 0 | -1 | Very low |
| Complication  9 studies (1466) | RCT and Non-RCT | 0 | -1 | -1 | 0 | 0 | 0 | 0 | Low |

Rate down if:

1. Data from a mixture of study designs.
2. ≥1 study with high risk of bias.
3. Heterogeneity was based on the extent of overlap of confidence intervals and I^2^ test (>50 %).
4. > 25 % of studies had a poor representation of outcome (when definitions of the outcome varied) or population (non-general population).
5. Total sample size < 500 participants.
6. Publication bias was based on the funnel plot and Egger’s test (*Unclear for outcomes with less than 10 studies included), the sample size of the included studies, ponsorship and/or conflict of interest report.
